# Synthesis, *in vitro* and *in silico* studies of a novel chrysin-ferrocene Schiff base with potent anticancer activity *via* G1 arrest, caspase-dependent apoptosis and inhibition of topoisomerase II

**DOI:** 10.1080/14756366.2025.2501377

**Published:** 2025-05-21

**Authors:** Mohammed Khaled Bin Break, Siddique Akber Ansari, Ahmed A. Katamesh, Najah Albadari, Maali D. Alshammari, Hamad M. Alkahtani

**Affiliations:** a Department of Pharmaceutical Chemistry, College of Pharmacy, University of Ha’il, Ha’il, Saudi Arabia; b Medical and Diagnostic Research Centre, University of Ha’il, Ha’il, Saudi Arabia; c Department of Pharmaceutical Chemistry, College of Pharmacy, King Saud University, Riyadh, Saudi Arabia; d Department of Pharmaceutics, College of Pharmacy, University of Ha’il, Ha’il, Saudi Arabia

**Keywords:** Cancer, chrysin-ferrocene, topoisomerase, tubulin, molecular dynamics

## Abstract

A novel chrysin-ferrocene Schiff base (CFSB) was synthesised as a potential anticancer agent. CFSB demonstrated high cytotoxicity against cancer cells with HepG2 (liver) being the most susceptible (IC_50_ = 3.11 µM). The compound was less toxic towards normal MRC5 cells and exhibited ∼5-fold selectivity towards most cancer cells. CFSB caused G1-phase arrest, induced caspase-dependent apoptosis by increasing Bax/Bcl2 ratio and reduced metastasis by decreasing MMP9 in HepG2. Furthermore, CFSB was inactive against CDK2, EGFR, TrkA and VEGFR, but it strongly inhibited topoisomerase II (IC_50_ = 20 µM) with potency comparable to etoposide (IC_50_ = 15 µM), while weak inhibition was observed against tubulin (IC_50_ = 76 µM). DFT calculations revealed that CFSB had desirable reactivity, while docking indicated high binding affinity with topoisomerase II. Molecular dynamics and MM-GBSA analyses showed that CFSB-topoisomerase II complex was stable with favourable binding energies, while *in silico* ADMET studies showed drug-like properties for CFSB.

## GRAPHICAL ABSTRACT

**Figure:**
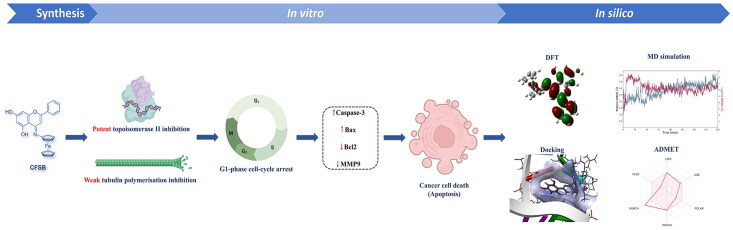

## Introduction

Cancer is a disease characterised by uncontrolled proliferation of transformed cells, while its heterogenous nature poses a great challenge towards its treatment. The disease remains a leading cause of death globally, with nearly 20 million new cancer cases and 9.7 million cancer deaths reported in 2022, while one in five individuals are expected to develop cancer in their lifetime. Projections indicate that new cancer cases could reach 35 million by 2050, emphasising the need for increased investment in prevention, control and treatment strategies^1^. The anticancer treatments that are currently available often suffer from limited potency and high toxicity, which highlights the importance of conducting further research to develop more efficient anticancer drugs. Natural products continue to be a valuable source of lead compounds for cancer drug discovery and development due to their chemical diversity and unique molecular properties^2^. Several approved anticancer drugs have been derived from natural products; such as the vinca alkaloids, taxanes and camptothecin analogues, while the unique and privileged structures of natural products have been utilised for synthesising a wide variety of effective and novel anticancer agents^3^.

**Figure 1.**
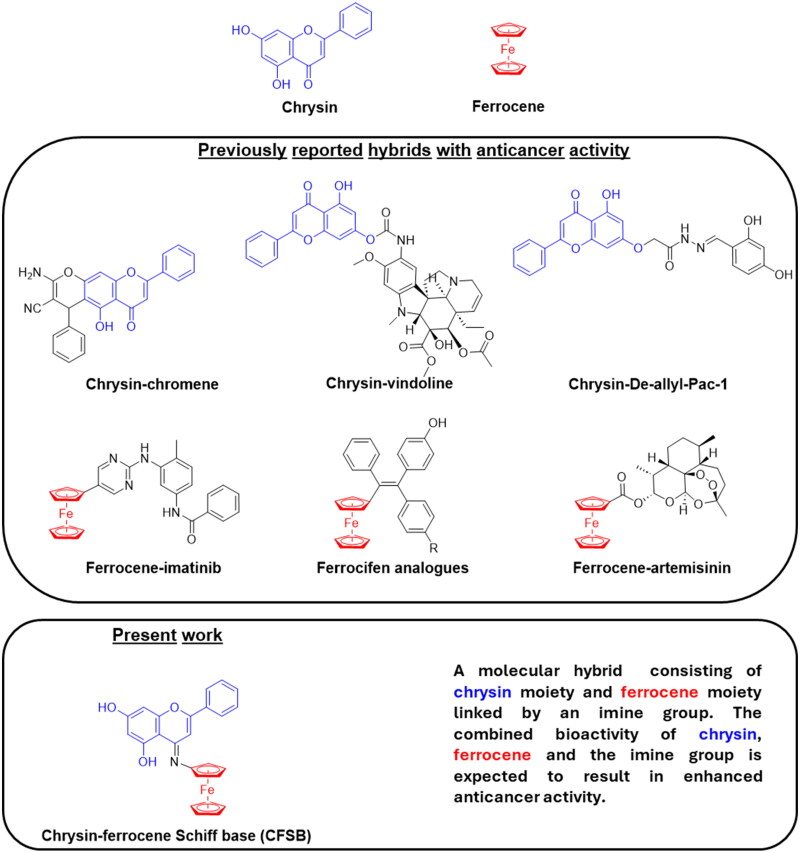
Chemical structures of selected anticancer chrysin and ferrocene hybrid analogues reported previously and of CFSB in the present study.

Chrysin, a naturally occurring flavone found in plants, honey and propolis, has garnered significant attention for its diverse pharmacological activities, particularly its anticancer properties. It has been shown to inhibit cancer growth through various mechanisms, including apoptosis induction *via* caspase activation and Bcl2 inhibition, PI3K/AKT/mTOR and MAPK/ERK pathway modulation in addition to angiogenesis inhibition *via* VEGF and MMP9 inhibition^4–7^. It is crucial to note that chrysin was found to exert its cytotoxic activity against cancer cells in a selective manner, without significantly affecting normal cells^4–5^. Furthermore, chrysin-based molecular hybrids were found to possess potent cytotoxic activity, and such hybrids include chrysin-chromene hybrids which demonstrated high anticancer potential against specifically K562 cells with IC_50_ value of 6.41 µM, in addition to chrysin-vindoline hybrids which were evaluated against a panel of 60 human cancer cell lines and found to exert potent activity with low micromolar GI_50_ values. Chrysin-De-allyl-Pac-1 is another chrysin hybrid which demonstrated high *in vitro* anticancer activity against the triple negative breast cancer cell line MDA-MB-231 with IC_50_ values as low as 5.98 µM^8–10^ (Figure 1).

Ferrocene, an organometallic compound with an iron centre sandwiched between two cyclopentadienyl rings, is another chemical compound that emerged as a versatile scaffold for anticancer drug development. The anticancer activity of its analogues is thought to arise from their unique properties; including structural diversity, stability, low toxicity, and redox activity^11^. Ferrocifen is a ferrocene derivative involving the tamoxifen moiety and was found to overcome multidrug resistance in cancer cells, in addition to possessing high cytotoxic activity with IC_50_ values as low as 0.02 µM against MDA-MB-231 and PC-3 cancer cell lines. Molecular hybrids such as ferrocene-artemisinin were found to exert potent cytotoxic activity against leukemic cells, such as CEM/ADR5000 cells with IC_50_ values at the nanomolar level (130 nM), while other ferrocene-trioxanes demonstrated high activity against breast and ovarian cancer cells, with mechanisms of action that involve synergistic effects with paclitaxel and enhancement of immunogenic cell death. Ferrocene-tyrosine kinase inhibitor hybrids were also found to possess high cytotoxic activity against bcr–abl positive cancer cell lines, with ferrocene-imatinib showing significantly high activity against LAMA-84 leukemic cell lines with an IC_50_ of 0.9 µM (Figure 1).^12–15^

Molecular hybridisation is considered a relatively modern strategy in rational drug design, and it involves combining two or more bioactive compounds or pharmacophoric moieties *via* linkers to produce a single compound with superior bioactivity. The resulting hybrid would often be multi-targeted which would make it highly active against diseases that possess a multifactorial nature, such as cancer^16^. Furthermore, molecular hybridisation has resulted in a number of FDA approved anticancer drugs, such as the carbazole hybrid alectinib, the quinoline hybrid cabozantinib and the hydroxamic acid hybrid Panobinostat^17^. Therefore, the anticancer potential that was demonstrated by chrysin-based and ferrocene-based analogues, more specifically hybrids, encouraged us to synthesise and investigate the first novel chrysin-ferrocene molecular hybrid. It is expected that the combined anticancer effects of the chrysin and ferrocenyl moieties would result in a highly effective anticancer agent. The resulting hybrid would most likely be multi-targeted as it would include the targets of both chrysin and ferrocene which should be effective in the case of cancer. It was suggested to link both bioactive moieties *via* an imine (C = N) linker to form a Schiff base compound. The imine group was chosen as a suitable linker because studies have proven it to be a critical pharmacophore in anticancer drug development, demonstrating diverse mechanisms of action and enhanced therapeutic potential when incorporated into synthetic complexes or natural products. Recent studies highlighted its role in DNA interaction, redox modulation, and targeted protein inhibition across multiple cancer types^18^^,^^19^.

Herein, we report for the first time, the synthesis and detailed anticancer potential of a novel chrysin-ferrocene Schiff base (CFSB) molecular hybrid (Figure 1). The present study involved the initial assessment of the compound’s cytotoxic activity *via* screening it against HepG2 (liver), HCT116 (colon), A549 (lung) and MCF7 (breast) cancer cells, followed by mechanism of action studies and enzyme inhibitory assays. Finally, molecular docking, molecular dynamics simulation and DFT calculations were conducted in order to further analyse CFSB’s binding with its target and provide a plausible explanation for its observed cytotoxic activity.

## Materials and methods

### Chemistry

#### General

Reagents of analytical grade were used as received from their commercial sources. Chrysin (99% purity) was obtained from Shanghai Yuanye Bio-Technology Co. Ltd. (Shanghai, China), while aminoferrocene was purchased from TCI (Japan). Melting points were obtained *via* an SMP100 Stuart apparatus and FTIR spectra were obtained using Nicolet iS10 FTIR spectrometer (Thermo Scientific, USA).^1^H NMR spectral analysis was performed using a Bruker FT-NMR 500 MHz, while ^13^C NMR analysis was conducted at 125 MHz. HRMS analysis was performed using a SCIEX TripleTOF 5600+ system equipped with a Duo-Spray^™^ source operating in the electrospray ionisation (ESI) positive mode (AB SCIEX, Concord, Canada). Analyst TF 1.7.1 (AB SCIEX, Concord, Canada) software was used for further analysis of the HRMS spectra. HPLC analysis was conducted using a Shimadzu Prominence (Shimadzu, Japan) LC-20AD Liquid Chromatograph pump equipped with a BDS HYPERSIL C18 column (5 µm, 150 mm x 4.6 mm). The HPLC system also consisted of a Shimadzu SPD-M20A detector, SIL-20AC autosampler, DGU-20A5R degasser and a CTO-20AC column oven. The method involved using gradient elution of an acetonitrile/water mobile phase system under a flow rate of 1.25 ml/min for 30 min. TLC was conducted using silica gel aluminium plates (Sigma-Aldrich) and a hexane/ethyl acetate mobile solvent at a ratio of 1:1.

#### Chrysin

IR (KBr, cm^−1^): 3081, 2628, 1651; ^1^H NMR (500 MHz, DMSO-*d_6_*, *δ*(ppm)): 12.83 (1H, 5-OH), 10.93 (1H, 7-OH), 8.06 (2H, dd, *J* = 8.1 Hz and 3.6 Hz, H-2′, 6′), 7.60 (3H, m, H-3′, 4′, 5′), 6.97 (1H, s, H-3), 6.52 (1H, d, *J* = 2.7 Hz, H-8), 6.23 (1H, d, *J* = 2.6 Hz, H-6); ^13^C NMR (125 MHz, DMSO-*d_6_*, *δ*(ppm)): 182.3 (C-4), 164.9 (C-7), 163.6 (C-2), 161.9 (C-5), 157.9 (C-9), 132.4 (C-4′), 131.2 (C-1′), 129.6 (C-3′, 5′), 126.9 (C-2′, 6′), 105.6 (C-3), 104.4 (C-10), 99.5 (C-6), 94.6 (C-8).

#### Synthesis of cyclopenta-2,4-dien-1-yl(5-((5,7-dihydroxy-2-phenyl-4H-chromen-4-ylidene)amino)cyclopenta-2,4-dien-1-yl)iron (CFSB)

Chrysin (145 mg, 0.57 mmol) was dissolved in methanol and left to stir and heat under reflux for 15 min. An equimolar amount of aminoferrocene (113 mg, 0.56 mmol) was then added to the chrysin mixture followed by the addition of drops of AcOH. The reaction was left to heat under reflux overnight until the yellowish solution turned dark red. The reaction was quenched by saturated NaHCO_3_ and extracted with EtOAc, while the organic layer was then dried over anhydrous MgSO_4_. Finally, the filtrate was concentrated *in vacuo* to afford a dark red solid.

Yield: 0.23 g (95%); mp: 196 °C; IR (KBr, cm^−1^): 3433, 3080, 1714; ^1^H NMR (500 MHz, DMSO-*d_6_*, *δ*(ppm)): 12.79 (1H, 5-OH), 9.89 (1H, 7-OH), 8.00 (2H, H-2′, 6′), 7.54 (2H, H-3′, 5′), 6.93 (1H, H-4′), 6.66 (1H, H-3), 6.49 (1H, H-8), 6.19 (1H, H-6), 5.94 (2H, H-13, 16), 5.75 (2H, H-14, 15), 4.24 (5H, Cp’); ^13^C NMR (125 MHz, DMSO-*d_6_*, *δ*(ppm)): 182.4 (C-4), 165.0 (C-7), 163.7 (C-2), 161.9 (C-5), 158.0 (C-9), 132.5 (C-4′), 131.2 (C-1′), 129.6 (C-3′, 5′), 126.9 (C-2′, 6′), 108.6 (C-12), 106.5 (C-13, 16), 105.7 (C-3), 104.4 (C-10), 99.5 (C-6), 94.6 (C-8), 69.1 (C-14, 15), 60.2 (Cp’); HRMS calculated for C_25_H_19_FeNO_3_ [M]^+:^ 437.0709, found: 437.0941; HRMS calculated for C_25_H_20_FeNO_3_ [M + H]^+^: 438.0787, found: 438.0978; Purity: 96.6% (HPLC, R_t_ = 4.309 min); *R_f_* (TLC): 0.84.

### Cell culture

Liver cancer (HepG2), colon cancer (HCT116), lung cancer (A549), breast cancer (MCF7) and normal lung (MRC5) cells were obtained from ATCC (Rockville, USA). Cells were cultured in RPMI-1640 enriched with 10% foetal calf serum and 50 µg/ml gentamycin. They were incubated at 37 °C in a humidified environment containing 5% CO_2_ and passaged 2–3 times/week.

### Cell viability assay

The MTT assay was conducted as previously reported^20^. Cell lines were seeded at 5x10^4^ cells/well and incubated for 24 h. CFSB was added in twelve concentrations, alongside vehicle controls and vinblastine sulphate as a positive control. After 72 h of incubation, cell viability was assessed using the MTT assay. Media was replaced, followed by the addition of MTT solution. DMSO was later added and optical density was measured at 590 nm using a microplate reader (TECAN, USA). IC_50_ values were calculated using Graphpad Prism (San Diego, USA). The experiment was repeated three times.

### Cell cycle analysis

Cell cycle analysis was conducted using the CycleTEST^™^ PLUS DNA Reagent Kit (Becton Dickinson Immunocytometry Systems, USA) as previously reported^21^. HepG2 cells were treated with CFSB at its IC_50_ for 72 h, and were then stained with propidium iodide according to the kit’s protocol and analysed using a cytometer. The cell cycle distribution was then determined using CellQuest software (Becton Dickinson Immunocytometry Systems, USA). The experiment was repeated three times.

### Apoptosis annexin V-FITC assay

Apoptosis assay was performed as previously described^21^. HepG2 cells were grown to a confluent monolayer and treated with CFSB at its IC_50_ concentration for 72 h. After treatment, cells were harvested, washed twice with PBS, and suspended in binding buffer. Both treated and untreated cells were then incubated with FITC-Annexin V for 40 min at 4 °C, washed, and resuspended in binding buffer containing DAPI. Finally, cells were analysed using a BD FACS Calibur flow cytometer (BD Biosciences, San Jose). The experiment was repeated three times.

### Caspase-3, Bax and Bcl2 ELISA assay

ELISA assay was conducted as previously reported^22^. HepG2 cells were grown to form a confluent monolayer and then exposed to CFSB at its IC_50_ concentration for 72 h. The cells were then harvested using trypsinisation with 0.25% trypsin, collected after centrifugation for 5 min, and washed with PBS before being suspended in binding buffer. The cells were finally analysed for markers of apoptosis, namely, caspase-3 Bax and Bcl2, using ELISA colorimetric kits (Neogen, Lexington, KY) as per the manufacturer’s instructions.

### MMP9 ELISA assay

MMP9 assay was conducted as previously reported^22^. Cells were seeded at a density of 1x10^5^ cells/ml one day prior to the experiment. HepG2 cells were maintained in RPMI-1640 containing 2% FBS, either with or without CFSB. Following a 72 h incubation period, the cells were harvested using trypsin, resuspended in 2 ml of culture medium, and centrifuged at 1500 rpm and 4 °C for 10 min. The cell pellet was then washed three times with PBS. Subsequently, the pellet was mixed with a solution consisting of 20 mM potassium phosphate buffer and a protease inhibitor. The cells were sonicated using a Virsonic^®^ ultrasonic cell disruptor for 10 min in cold saline, followed by centrifugation at 5000 rpm for 5 min at 4 °C. The resulting supernatants were collected, and MMP9 concentrations were measured using ELISA kits (Neogen, Lexington, KY) according to the manufacturer’s instructions.

### Enzyme inhibition assays

CDK2 kinase assay was performed using the CDK2 kinase assay kit (BPS Bioscience, USA) as previously described^23^. The EGFR kinase assay was performed in 96-well plates coated with PGT (poly l-glutamic acid l-tyrosine, Sigma Aldrich, USA) using the EGFR kinase assay kit (BPS Bioscience, USA) and was conducted as previously described^24^. The VEGFR-2 kinase assay was carried out in 96-well streptavidin coated plate using recombinant human VEGFR-2/KDR ELISA kit as previously described^25^. The tropomyosin receptor kinases (TRK) inhibition assay was performed using TrkA assay kit (BPS Bioscience, USA) as per the manufacturer’s instructions. Topoisomerase II assay was performed using the human topoisomerase II relaxation assay kit (Inspiralis, UK) which involved relaxing supercoiled pBR322 DNA substrate by the enzyme. The relaxation assay was conducted as per the manufacturer’s instructions and as previously reported^26^.

### Tubulin polymerisation assay

The assay was performed using the tubulin polymerisation assay kit (Cytoskeleton, Inc, USA) as per the manufacturer’s instructions and as previously reported^27^. In brief, tubulin was mixed with different concentrations of CFSB or combretastatin in buffer (80 mM PIPES, 0.5 mM EGTA, 2 mM MgCl_2_, pH 6.9) containing 1 mM GTP and 15% glycerol. Polymerisation dose-response curves were obtained by measuring changes in fluorescence intensity at each compound concentration. Fluorescence was measured at an excitation of 360 nm and emission of 450 nm using a Tecan-spark reader.

### DFT calculations

DFT experiments were performed following a previously reported protocol^28^. DFT calculations were conducted using Gaussian 09 W, while visualisation was conducted using GaussView. CFSB geometrical optimisation was performed using the B3LYP method with 6–311 G+ (d, p) basis set in DMSO using the SMD solvation model. HOMO-LUMO energies were analysed for CFSB at the 6–311 G+ (d, p) level in DMSO.

### Molecular docking

The optimised CFSB structure *via* DFT calculations was used for molecular docking. The Schrodinger suite was used for docking based on a previously reported protocol^29^. The relevant PDB file for the target protein was imported into Maestro software and the protein was prepared for docking by removing water molecules, adding hydrogens and missing residues, and minimising its energy *via* the OPLS4 force field. Grids centred around the proteins’ co-crystallised ligand were generated using Glide, while CFSB optimised structure was further processed using LigPrep. Finally, flexible CFSB-protein docking experiments with single precision mode (SP) were performed using Glide.

### MD simulations

Molecular dynamics simulation was performed for 200 ns for CFSB-topoisomerase II and etoposide-topoisomerase II complexes using Desmond, a Schrodinger Package. The previously obtained docking results were used to further conduct molecular dynamic (MD) simulations for the CFSB-topoisomerase II complex, while the available crystal structure (PDB ID: 3QX3) was used for the etoposide-topoisomerase II complex. MD simulation was performed as previously reported^30^. Briefly, complexes were pre-processed using the protein preparation wizard. The NPT ensemble with a temperature of 300 K and a pressure of 1 atm was applied and the simulation was run for a total duration of 200 ns. The OPLS2005 force field parameters were used. The orthorhombic periodic box was set 10 Å away from the protein’s outer surface, while the TIP3P model was used to describe water molecules. Charges on the box were neutralised *via* counter ions, followed by the addition of 0.15 M NaCl. Hydrogen bonds and electrostatic interactions were managed using the SHAKE algorithm and the particle mesh Ewald (PME) summation method, respectively. Trajectories were saved at 100 ps intervals for analysis, and simulation’s stability was assessed by continuously monitoring root mean square deviation (RMSD) of the protein and ligand. Finally, simulation results were analysed *via* the Simulation Interaction Diagram tool in Desmond.

### Prime MM-GBSA calculations

MM-GBSA calculations were performed according to a previously mentioned protocol^30^. The CFSB-topoisomerase II and etoposide-topoisomerase II docked complexes were energetically minimised *via* the Prime module present in the Schrodinger software. This was followed by implementing the molecular mechanics generalised Born surface area (MM-GBSA) to measure the binding free energy of the complexes. For the calculation, a Desmond trajectory file is divided into frames, and the MM-GBSA calculations are then conducted on each frame resulting in an average binding energy as the output.

### In silico ADMET studies

ADMET studies were conducted on CFSB using SwissADME^31^, pkCSM^32^, PredPS^33^ and SMARTCyp^34^ Software. The parameters selected for analysis were based on a previously reported protocol^35^. Physicochemical and drug-likeness studies, bioavailability radar chart and BOILED-Egg graph were all analysed using SwissADME. Parameters related to toxicity and CYP450 inhibition were analysed *via* pkCSM, while parameters related to metabolic stability were investigated using PredPS.

### Statistical analysis

Data were processed using GraphPad Prism and presented as mean ± standard deviation (SD). Mean differences were assessed using two-way ANOVA, followed by multiple comparison tests and statistical significance was denoted as *p < 0.05 vs. control, **p < 0.01 vs. control, ***p < 0.001 vs. control.****p < 0.0001 vs. control.

## Results and discussion

### Chemistry

**Scheme 1.**
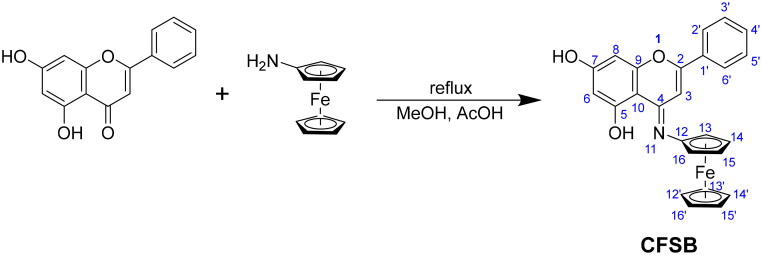
Synthesis of CFSB.

The novel synthetic chrysin-ferrocene Schiff base (CFSB) has been synthesised according to Scheme 1. The reaction was straightforward and involved reacting equimolar amounts of chrysin and aminoferrocene in methanol, in the presence of acetic acid as a catalyst. The reaction was heated under reflux overnight and a gradual change in colour from orange to dark reddish-brown was observed as the reaction proceeded, while the product was finally obtained after a simple work-up using saturated NaHCO_3_. TLC was used in order to confirm completion of the reaction.

CFSB was thoroughly characterised in order to confirm that the intended compound has been obtained. CFSB’s FTIR spectrum (Figure S1) showed a characteristic peak attributed to the newly formed imine bond (C = N) at 1714 cm^−1^. This peak was missing from chrysin’s spectrum (Figure S6), indicating the successful formation of CFSB. It is also interesting to note that chrysin’s OH groups showed a peak at 3080 cm^−1^, but this wavenumber is much lower than the typical value of 3400 cm^−1^ and it is mostly due to intramolecular hydrogen bonding with the carbonyl group, in addition to the highly conjugated nature of chrysin that leads to redistribution of electron density^36^^,^^37^. However, CFSB showed a peak at 3433 cm^−1^ assigned to OH of chrysin’s moiety, which indicates that the ferrocenyl moiety may have redistributed the electron density across the whole structure resulting in a different vibrational frequency for OH than that of pure chrysin. This phenomenon seems to have affected 5-OH only, as the peak for 7-OH was still present at 3080 cm^−1^. Therefore, the change in 5-OH wavenumber for CFSB relative to chrysin, is further evidence that the Schiff base has been synthesised successfully.

**Figure 2.**
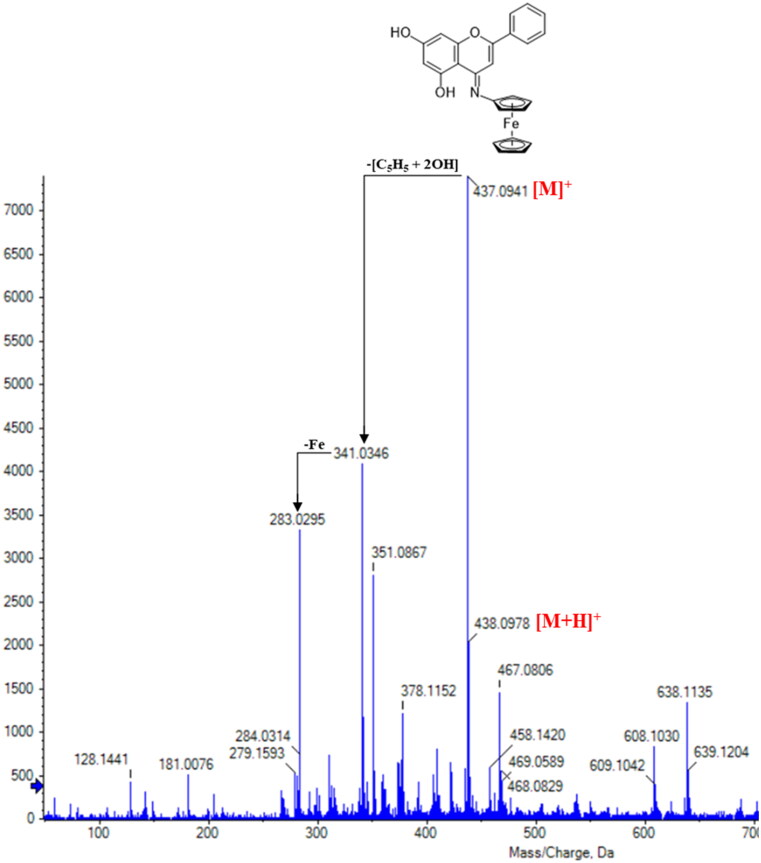
ESI-HRMS analysis for CFSB.

CFSB was also characterised *via* high-resolution mass spectrometry (HRMS), whereby a molecular ion peak of relatively high abundance was observed at 437.0941 [M]^+^, followed by another peak adduct at 438.0978 [M + H]^+^ (Figures 2 and S2). These two peaks confirmed that the desired compound has been successfully obtained. The peak at 341.0346 corresponds to a fragment resulting from the loss of a cyclopentadienyl ligand and two hydroxyl groups, while the further loss of Fe (II) resulted in a fragment ion that is most probably behind the peak at 283.0295. It is crucial to note that cleavage of the Fe-cyclopentadienyl bond in ESI-MS is a characteristic fragmentation pathway for most ferrocene analogues^38^, and this could be a further indicator for the formation of our intended ferrocene derivative. The peaks that are greater than [M + H]^+^ are thought to have resulted from the formation of adduct ions and aggregates, for instance, the peaks at 467 and 469 most probably resulted from the adducts [M + CH_3_OH-2H]^+^ and [M + CH_3_OH]^+^, respectively.

^1^H NMR spectrum of CFSB (Figure S3) and chrysin (Figure S7) have been included in the Supplementary Material File. NMR spectral analysis of CFSB showed broader peaks with lower intensities, especially for nuclei that are in close proximity to Fe (II), and this is thought to arise as a result of the paramagnetic nature of Fe (II). Ferrocene is actually diamagnetic, however, it may change to paramagnetic depending on the nature and structure of the ligands^39^^,^^40^, and it seems in our case that introduction of chrysin moiety to the cyclopentadienyl ligand resulted in a paramagnetic compound, as shown by its NMR spectra. The effect of paramagnetism on NMR peak broadening, loss of peak splitting patterns, suppression of peak intensities and inaccurate peak integration has been extensively studied and reported^41^^,^^42^. However, in our case, the spectra’s clarity was adequate to at least enable CFSB structure elucidation. ^1^H NMR spectrum (Figure S3) showed peaks that are characteristic to the chrysin moiety, whereby the peaks between 7–8 ppm were attributed to the benzene ring protons of the structure, while the two singlets at 9.89 and 12.79 ppm were assigned to hydroxyl groups 7-OH and 5-OH, respectively. Methine proton H-3 was assigned the peak at 6.66 ppm. There were also peaks corresponding to the ferrocenyl moiety of the compound. Protons of the unsubstituted cyclopentadienyl ligand (Cp’-H) were assigned the peak at 4.21 ppm, while protons of the other substituted cyclopentadienyl ligand were slightly deshielded due to the adjacent electronegative nitrogen atom resulting in peaks at around 5.8 ppm. It is highly crucial to note that the peaks’ splitting patterns were challenging to observe due to peak broadening resulting from paramagnetism, and this effect further proves the formation of the intended compound.

^13^C NMR spectrum of CFSB (Figure S4) and chrysin (Figure S8) have been included in the Supplementary Material File. ^13^C NMR spectrum (Figure S4) showed peaks at around 130 ppm corresponding to the carbon atoms of the benzene ring, while C-OH of the dihydroxyphenyl group (C-5 and 7) showed 2 peaks at around 160 ppm. The imine (C = N) carbon was assigned the peak at 182.35 ppm, while the presence of other peaks attributed to the chrysin moiety further confirmed the suggested structure of CFSB. As for the structure’s ferrocenyl moiety, the unsubstituted cyclopentadienyl carbon atoms (Cp’) were assigned the peak at 60.24 ppm, however, the other cyclopentadienyl ring experienced extensive deshielding resulting in a peak for C-12 at 108.58 ppm, while the adjacent pair of carbon atoms (C-13 and 16) were assigned the peak at 106.45 ppm and the last carbon atoms of the ring (C-14 and 15) gave rise to the peak at 69.08 ppm. Therefore, the presence of peaks for both the chrysin and ferrocenyl moieties further confirm the successful synthesis of CFSB.

Finally, the purity of CFSB was confirmed *via* HPLC and was found to be 96.6% (Figure S5).

### Biological activity

#### CFSB demonstrated cytotoxic activity against HepG2, HCT116, A549 and MCF7 cancer cells in a dose-dependent manner

Studies have clearly proven the anticancer potential of chrysin and ferrocene, as well as their analogues. In this study, we aimed to produce a chrysin-ferrocene molecular hybrid with superior anticancer activity that is suggested to result from the combined biological effects of the chrysin and ferrocenyl moieties. Moreover, the two moieties were linked *via* an imine bond, which itself possesses cytotoxic activity. Therefore, the synthetic chrysin-ferrocene Schiff base (CFSB) was screened against HepG2 (liver), HCT116 (colon), A549 (lung) and MCF7 (breast) cancer cells, and its cytotoxic activity was determined using the MTT assay.

**Figure 3.**
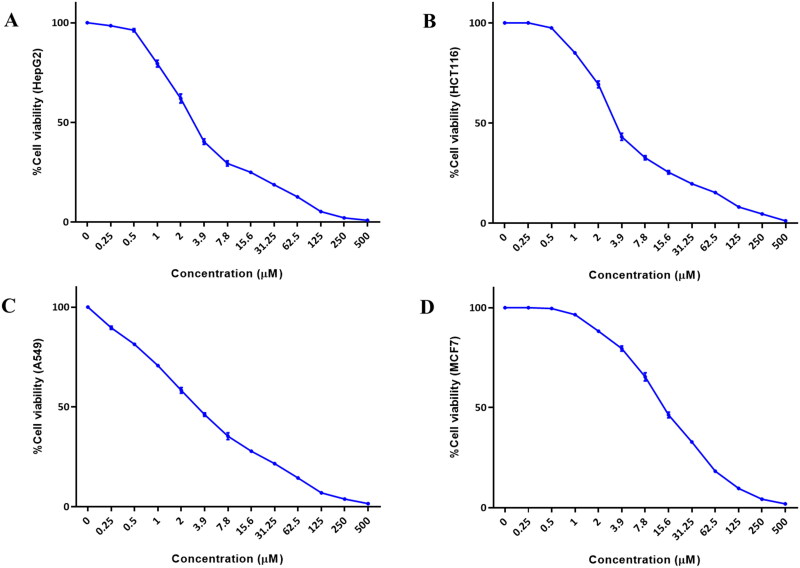
Representative dose-response profiles showing cytotoxic activity of CFSB against (a) HepG2; (b) HCT116; (c) A549; (d) MCF7 cancer cells. Points refer to mean values of three experiments.

Results of the assay demonstrated that CFSB exerted cytotoxic activity against all the investigated cancer cells with almost similar potency, except in the case of MCF7 which was significantly less affected by the compound (Figure 3; Table 1). CFSB exerted its highest cytotoxic activity against HepG2 cells, followed by A549, HCT116 and finally MCF7, with IC_50_ values of 3.11 µM, 3.35 µM, 3.47 µM and 14.08 µM, respectively. It is interesting to note that CFSB demonstrated comparable cytotoxic activity to the standard drug doxorubicin across all cell lines except MCF7, while higher activity than vinblastine was observed against A549 cells, which highlights CFSB’s potential as a potent anticancer agent. Since HepG2 cells were the most responsive cells to CFSB treatment, further mode of action studies would be performed on them.

**Table 1.** Cytotoxic activity of CFSB against HepG2, HCT116, A549 and MCF7 cancer cells.

	IC_50_ (µM)^a^			
Compound	HepG2	HCT116	A549	MCF7
CFSB	3.11 ± 0.41	3.47 ± 0.69	3.35 ± 0.27	14.08 ± 0.96
Vinblastine sulphate	0.89 ± 0.10	1.75 ± 0.19	4.15 ± 0.31	2.27 ± 0.25
Doxorubicin	2.65 ± 0.23	3.16 ± 0.38	3.51 ± 0.45	3.67 ± 0.62

^a^ IC_50_ values are reported as the mean (IC_50_ **±** SD) of three experiments.

#### CFSB shows lower toxicity towards MRC5 healthy lung cells and more selectivity towards HepG2, HCT116 and A549 cancer cells

Potential anticancer drugs should ideally demonstrate high cytotoxic activity against cancer cells without resulting in any harmful effects towards normal healthy cells. The toxicity of CFSB towards normal cells was assessed by screening it against normal, healthy MRC5 lung cells, followed by calculating its selectivity index (SI) to evaluate CFSB’s selectivity towards cancer cells (Table 2). Results demonstrated that CFSB was approximately 5-fold more selective towards cancer cells relative to normal, healthy cells, with the only exception being against MCF7 breast cancer cells, whereby no significant selectivity was observed for the compound towards this specific cancer cell line. Therefore, it can be deduced from the assay that CFSB shows high selectivity for HepG2, HCT116 and A549 cancer cells relative to normal MRC5 cells, and that indicates less toxic side effects are expected to occur at CFSB’s therapeutic dose.

**Table 2.** Cytotoxic activity of CFSB against healthy MRC5 cells.

	IC_50_ (µM)^a^	Selectivity Index (SI)^b^			
Compound	MRC5	HepG2	HCT116	A549	MCF7
CFSB	16.96 ± 1.82	5.5	4.9	5.1	1.2

^a^ IC_50_ values are reported as the mean (IC_50_ **±** SD) of three experiments.

^b^ SI = (IC_50_ of MRC5)/(IC_50_ of cancer cell).

#### CFSB-induced G1-phase cell-cycle arrest in HepG2 cancer cells

**Figure 4.**
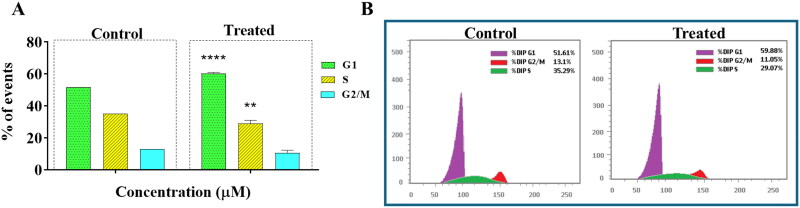
(a) Annexin V/PI apoptosis assay revealed that CFSB induced apoptosis in HepG2 cells after 72 h of treatment at IC_50_. The experiment was repeated three times. **p < 0.01 vs. control. (b) Representative images showing the apoptotic effects of CFSB on HepG2 cells.

Cell-cycle dysregulation is a main feature of several cancers leading to uncontrolled cell proliferation and tumour growth^43^. In the present study, HepG2 cells were treated with CFSB at its IC_50_ concentration followed by flow cytometric analysis, in order to investigate the compound’s potential to affect the cell-cycle of HepG2 cells (Figure 4). HepG2 was chosen for further analysis as it was the most cell line susceptible to CFSB treatment. Results showed that there was a significant accumulation of cells in the G1-phase after CFSB treatment (60.3% relative to 51.6% in the control group), accompanied by a significant decrease in the number of cells in the S-phase (29.0% relative to 35.3% in the control group), as well as the G2/M-phase (10.7% relative to 13.1% in the control group). Therefore, it can be deduced that CFSB induces G1-phase cell-cycle arrest in HepG2 cells.

#### CFSB-induced caspase-dependent apoptosis, increased Bax/Bcl2 ratio and decreased MMP9 expression level in HepG2 cancer cells

**Figure 5.**
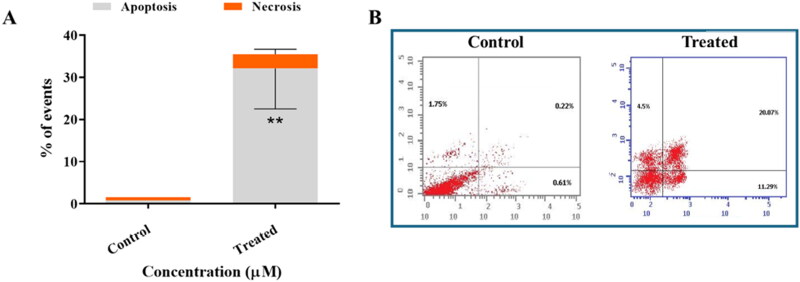
(a) Annexin V/PI apoptosis assay revealed that CFSB induced apoptosis in HepG2 cells after 72 h of treatment at IC_50_. Experiment was repeated three times. **p < 0.01 vs. control. (b) Representative images showing the apoptotic effects of CFSB on HepG2 cells.

Apoptosis is a crucial cellular process that involves programmed cell death, and is often a desirable outcome for agents that target cancer cells. Therefore, the apoptosis-inducing effects of CFSB were investigated using an annexin V/propidium iodide apoptosis assay, whereby HepG2 cells were treated with CFSB at its IC_50_ concentration for 72 h followed by the apoptosis assay (Figure 5). Results revealed that CFSB caused a significant increase in the percentage of apoptotic cells, with about 32% apoptotic cells after treatment relative to 0.83% in the control group. Moreover, there was a small insignificant increase in the percentage of necrotic cells after CFSB treatment, with about 3.4% necrotic cells after treatment relative to 1.8% in the control group.

Activation of caspases is considered to be a clear indication and hallmark of apoptosis, especially caspase-3 which is an executioner caspase involved in cleaving the majority of cellular substrates during apoptosis^44^. Induction of apoptosis could be further confirmed by investigating the expression level of apoptosis-related proteins, such as Bax and Bcl2. Bax is a pro-apoptotic protein that is responsible for caspase activation, however, its activity is often suppressed by the anti-apoptotic protein Bcl2^45^. The Bax/Bcl-2 expression ratio is a crucial determinant of the cell’s survival or death, whereby a high ratio promotes apoptosis, while a low ratio enhances cell survival^46^. Therefore, CFSB’s effect on the expression levels of these apoptosis-related proteins were investigated using an ELISA assay, after treatment of HepG2 cells. It was found that CFSB caused an increase in the expression level of caspase-3 and Bax, while a decrease in Bcl2 expression was found after treatment, which in turn resulted in a higher Bax/Bcl2 ratio relative to the untreated control group (Table 3). These results indicate that CFSB induced caspase-dependent apoptosis in HepG2 cells which corroborates the results of the annexin V/PI apoptosis assay.

**Table 3.** Effect of CFSB, at its IC_50_ concentration, on the expression levels of Bax, Bcl2, caspases-3 and MMP9 in HepG2 cancer cells.

Compound	Caspase-3 (ng/ml)	Bax (ng/ml)	Bcl2 (ng/ml)	Bax/Bcl2 ratio	MMP9 (pg/ml)
CFSB	132.12 ± 3.30	2.71 ± 0.18	3.40 ± 0.18	0.80	57.63 ± 3.30
Control	78.35 ± 1.89	1.65 ± 0.13	6.41 ± 0.39	0.26	97.36 ± 2.72

Values are reported as the mean (IC_50_ **±** SD) of three experiments.

Matrix Metalloproteinase-9 (MMP9) plays a significant role in cancer development, progression, and metastasis. It degrades extracellular matrix components, facilitating tumour cell invasion and metastasis, in addition to promoting the formation of new blood vessels resulting in angiogenesis^47^^,^^48^. This makes MMP9 inhibition a potential cancer treatment strategy. Our present study showed that CFSB decreased MMP9 expression level (**Table 3**), indicating that it suppresses cancer cell metastasis and invasion.

#### CFSB showed no significant inhibitory activity against CDK2, EGFR, TrkA and VEGFR enzymes

Cyclin-dependent kinase 2 (CDK2) plays an essential role in cell cycle regulation and several other biological processes; such as DNA damage and replication, in addition to signal transduction. CDK2 inhibition has been proven to result in antitumour activity, which made it an attractive target for several anticancer agents^49^. It has been previously shown that the anticancer mode of action of chrysin and some ferrocenyl analogues involves CDK2 downregulation^50^^,^^51^.

Epidermal growth factor receptor (EGFR) is a tyrosine kinase receptor that is often involved in cell proliferation and survival, however, its aberrant activation is associated with various cancers, and this made it a well-established target for anticancer agents^52^. Tropomyosin receptor kinase A (TrkA) is another tyrosine kinase receptor that is usually overexpressed in most tumours resulting in tumour cell proliferation, invasion, angiogenesis and metastasis^53^^,^^54^. A chrysin analogue (CHM-04) has been found to exert its cytotoxic activity *via* EGFR inhibition, while chrysin itself has been reported to potentially bind with TrkA^55–57^. A further tyrosine kinase receptor is vascular endothelial growth factor receptor (VEGFR) which plays a crucial role in tumour angiogenesis, making it a key target for cancer therapy^58^. Chrysin was shown to downregulate the expression level of VEGFR, while some ferrocenyl analogues were found to specifically inhibit VEGFR2^59^^,^^60^.

**Figure 6.**
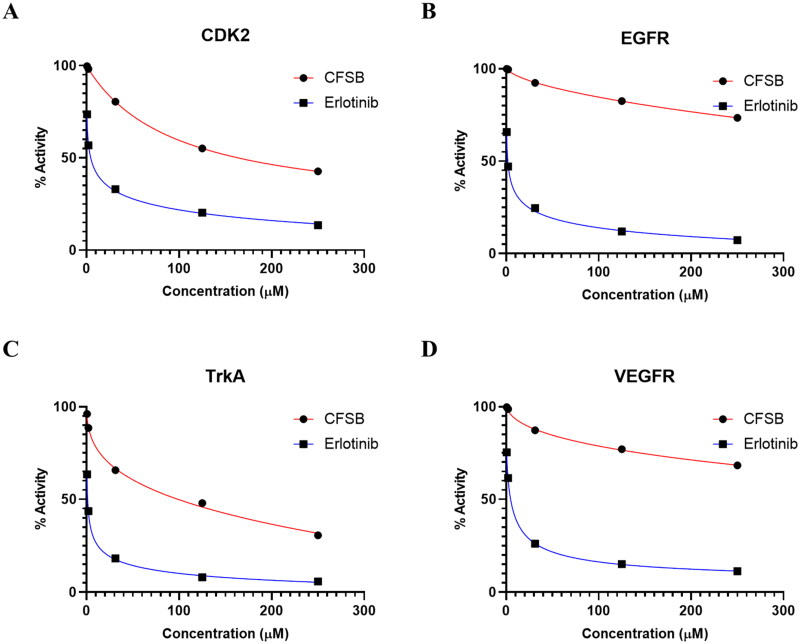
Dose-response curves demonstrating the inhibitory effect of CFSB against (a) CDK2, (b) EGFR, (c) TrkA, and (d) VEGFR.

Therefore, based on the activity of chrysin and/or ferrocenyl analogues against CDK2, EGFR, TrkA and VEGFR, in addition to these receptors’ involvement in cancer progression, it was decided to investigate the potential inhibitory activity of CFSB against these proteins. Results of the assays showed that CFSB possessed extremely weak activity against the investigated protein targets, indicating that the compound’s mode of action does not involve any of these receptors (Figure 6; Table 4).

**Table 4.** IC_50_ values of CFSB against CDK2, EGFR, TrkA and VEGFR.

IC_50_ (µM)^a^				
Compound	CDK2	EGFR	TrkA	VEGFR
CFSB	>100	>100	>100	>100
Erlotinib	4.26 ± 0.52	1.79 ± 0.37	1.25 ± 0.17	3.65 ± 0.51

^a^ IC_50_ values are reported as the mean (IC_50_ **±** SD) of three experiments.

#### CFSB exerts potent inhibitory activity against topoisomerase II but shows weak tubulin polymerisation inhibition

Nucleic acid processes, such as DNA replication, often result in knots and tangles that may impair cellular function. These lethal topological obstacles in the DNA structure are often regulated by topoisomerases^61^. There are two types of topoisomerases; topoisomerase I cleave single DNA strands, while topoisomerase II is an important nuclear enzyme that is often involved in breaking double-stranded DNA and relaxation of supercoiled strands in order to facilitate DNA replication and transcription. It was found that topoisomerase II is often highly expressed with a prolonged half-life in cancer cells, which made it a suitable and desirable target for cancer treatment^62^^,^^63^. Studies have previously shown that chrysin exerted its cytotoxic activity, at least in part, by potentially inhibiting topoisomerase II^64^^,^^65^. Furthermore, ferrocene analogues were also found to inhibit topoisomerase II^66^^,^^67^. Therefore, we reasoned that the synergistic effects of a chrysin-ferrocene hybrid such as CFSB would possess potent topoisomerase II inhibitory activity.

**Figure 7.**
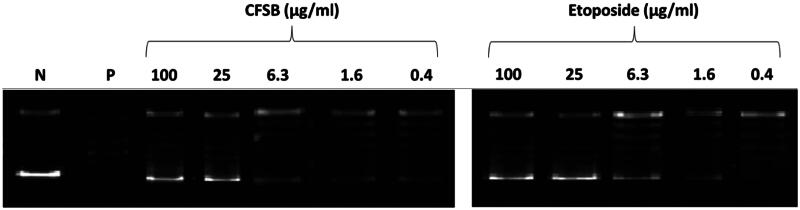
Gel electrophoresis image representing the relaxation potential of supercoiled DNA (pBR322) by topoisomerase II in the presence of CFSB and etoposide at different treatment concentrations. The N (Negative) lane represents supercoiled DNA only with no enzyme, while the P (Positive) lane represents supercoiled DNA with uninhibited topoisomerase II.

**Figure 8.**
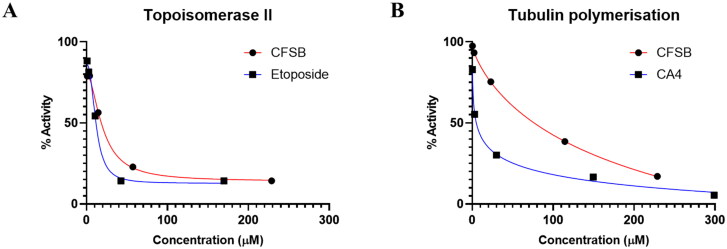
Dose-response curves demonstrating the inhibitory effect of CFSB against (a) topoisomerase II and (b) tubulin polymerisation.

The potential inhibition of topoisomerase II by CFSB was evaluated using human topoisomerase II assay. This assay involves measuring the ability of topoisomerase II enzyme to relax supercoiled DNA (pBR322)^68^. Results of the assay showed that CFSB inhibited topoisomerase II activity in a dose dependent manner and possessed inhibitory activity that was comparable to that of the standard drug, etoposide, with IC_50_ values of 20.07 µM and 14.89 µM, respectively (Figures 7 and 8; Table 5). These comparable IC_50_ values indicate the potent inhibitory activity of CFSB against topoisomerase II. It is important to note here that topoisomerase II inhibition by CFSB is thought to be the main reason behind the G1-phase cell-cycle arrest that was found to be induced by the compound, as studies have shown that topoisomerase II inhibition might also result in G1-phase arrest, and not only S or G2-phase arrest as previously thought^69^^,^^70^.

**Table 5.** IC_50_ values of CFSB against topoisomerase II and tubulin polymerisation.

IC_50_ (µM)^a^		
Compound	Topoisomerase II	Tubulin
CFSB	20.07 ± 0.96	76.39 ± 1.35
Etoposide	14.89 ± 0.75	–
Combretastatin A4	–	4.90 ± 0.19

^a^ IC_50_ values are reported as the mean (IC_50_ **±** SD) of three experiments.

Microtubules are cylindrical structures that form a crucial part of the cell’s cytoskeleton and are made up of α and β-tubulin heterodimers. These dimers polymerise to form microtubules which later disassemble to the heterodimers again resulting in a continuous tubulin-microtubule dynamic system. Microtubules are involved in a variety of essential cellular functions, such as cell division and movement. Anticancer agents such as taxanes disrupt the tubulin-microtubule dynamic system by stabilising microtubules, while drugs such as the *Vinca* alkaloids enhance depolymerisation of microtubes to α and β-tubulin^71^. Ferrocenyl analogues have been previously reported to exert their anticancer effect *via* inhibiting tubulin polymerisation^72^. Therefore, we reasoned that our ferrocenyl derivative, CFSB, could also inhibit tubulin polymerisation as part of its anticancer mode of action.

A tubulin polymerisation assay was used in order to assess the potential of CFSB as a tubulin polymerisation inhibitor. The assay involved observing the polymerisation of tubulin, isolated from porcine brain tissue, in the presence of CFSB. Changes in polymerisation were monitored *via* changes in fluorescence intensity. Results of the assay showed that CFSB inhibited tubulin polymerisation weakly when compared with the reference drug combretastatin A4 (CA4), with IC_50_ values of 76.4 µM and 4.9 µM, respectively (Figure 8; Table 5). Therefore, it seems that inhibition of tubulin polymerisation is not the main mechanism by which CFSB exerts its cytotoxic activity, however, it still seems to be a crucial part of its bioactivity along with topoisomerase II inhibition. In conclusion, CFSB exerts its cytotoxic activity *via* potent inhibition of topoisomerase II and weak tubulin polymerisation inhibition.

### In silico studies

#### Density functional theory (DFT)

**Figure 9.**
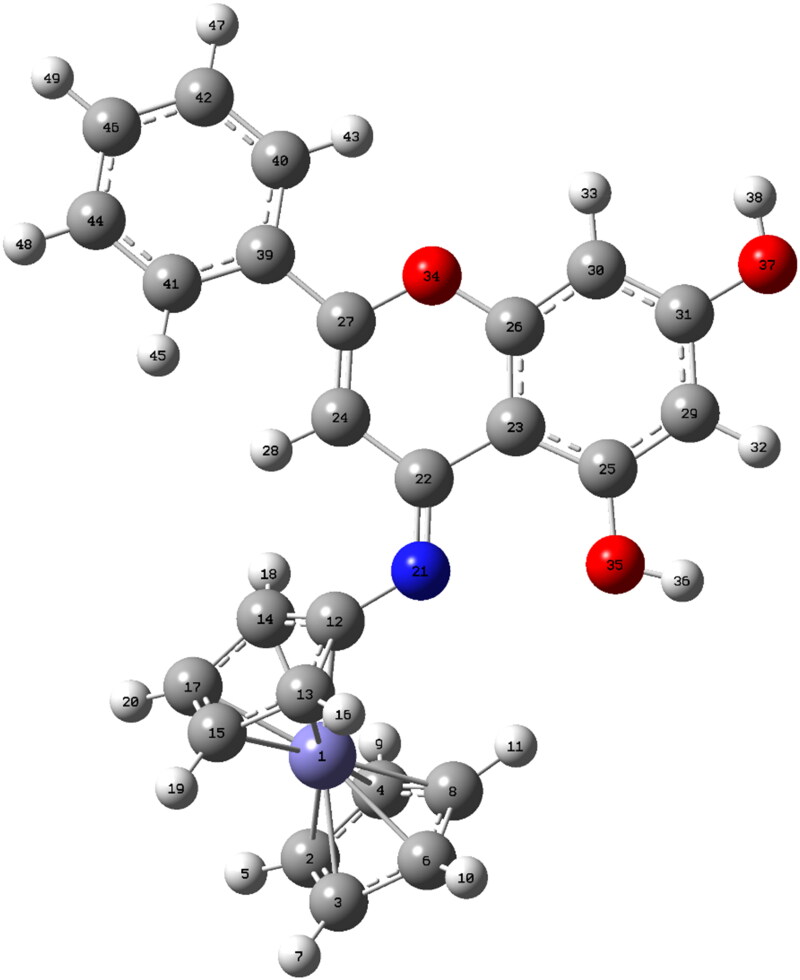
Optimised structure of CFSB obtained at B3LYP/6-311G+ (d,p).

The geometric structure of a compound is related to its stability and binding affinity with its target, which in turn affects its bioactivity^73^. The geometric structure of CFSB was optimised *via* DFT at B3LYP/6-311G+ (d, p) level, and shown in Figure 9, while the calculated geometric parameters have been shown in Table 6. DFT calculations were performed in DMSO solvent rather than the gas phase, as it was the solvent used in the previously mentioned bioassays of the study. The optimised structure had an electronic energy of −2508.578107 Hartree and a dipole moment of 5.876717 Debye.

**Table 6.** Selected theoretical geometric parameters for CFSB (Å, °).

Geometric parameters^a^	Theoretical (B3LYP)
Bond distance (Å)	
C22—N21	1.29903
C12—N21	1.39776
C24—C27	1.34896
C27—O34	1.36248
C26—O34	1.37031
C26—C30	1.39149
C31—O37	1.35930
C25—O35	1.35568
C13—Fe1	2.07210
C41—C44	1.39064
Bond angles (°)	
C44—C41—C39	120.5
C27—C24—C22	123.0
C27—O34—C26	120.5
C26—C23—C25	115.2
C29—C31—O37	117.2
C22—C21—N12	121.9
C12—C14—C17	108.3
C15—Fe1—C13	40.3
C29—C25—C35	119.2
Torsion angles (°)	
C27—C24—C22—N21	178.7
O34—C26—C22—N21	−179.5
C22—N21—C12—C15	−134.8
C13—Fe1—C6—C8	−122.0
O34—C26—C24—C22	179.5
C30—C31—C23—C25	−179.7
O34—C22—C8—C3	−138.1
C26—C24—C41—C42	24.5
C15—C12—C29—C24	−135.5

^a^ Atom numbering is based on Figure 8.

The optimised structure was further analysed in order to determine CFSB’s reactivity with its target receptor relative to its parent compound, chrysin, and that was accomplished by investigating the compounds’ frontier molecular orbitals (FMO), which include the HOMO and LUMO, whereby their analysis enables assessment of a compound’s reactivity. HOMO analysis aids in determining the ability to donate electrons, while LUMO analysis assesses the molecule’s ability to accept electrons, and such investigations are extremely crucial as they are strongly related to the molecule’s bioactivity^74^.

**Figure 10.**
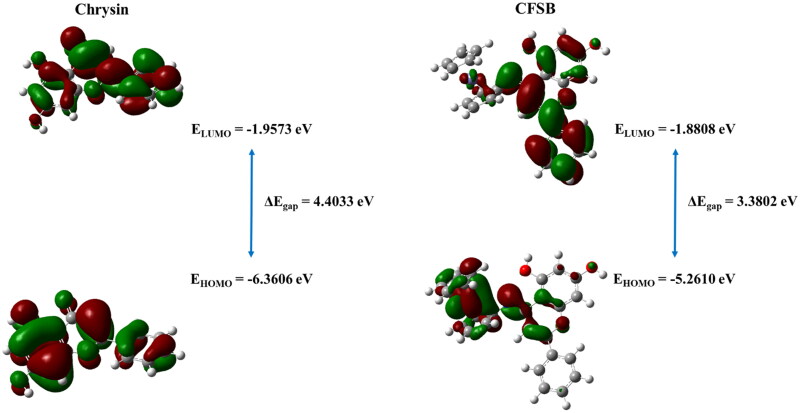
HOMO and LUMO energies of chrysin and CFSB at B3LYP/6-311G+ (d,p) level.

HOMO and LUMO energies for chrysin and CFSB have been shown in Figure 10. It can be seen, in the case of CFSB, that the LUMO molecular orbitals are mostly concentrated on the chrysin moiety of the structure, while HOMO orbitals are located around the ferrocene moiety. On the other hand, chrysin’s LUMO orbitals are slightly more concentrated at ring B of the structure, while HOMO orbitals are more concentrated at ring A. A molecule’s tendency to accept electrons is indicated by a lower LUMO energy (*E*_LUMO_), while a higher HOMO energy (*E*_HOMO_) reflects a molecule’s tendency to donate electrons^75^. However, a more accurate method to assess a molecule’s electrophilicity/nucleophilicity, as well as reactivity is *via* using global reactivity descriptors.

The following formulas may be used to calculate ionisation potential (IP) and electron affinity (EA):

(1) $$\text{EA}={-E}_{\text{LUMO}}$$

(2) $$\text{IP}={-E}_{\text{HOMO}}$$

Equations (1) and (2) are used to calculate absolute electronegativity (*χ*), chemical potential (*µ*), absolute hardness (*η*) and softness (*S*), as shown below^76^:

(3) $$\chi =‐\mu =\frac{1}{2}(\text{IP}+\text{EA})$$

(4) $$\eta =\frac{1}{2}(\text{IP}-\text{EA})$$

(5) S=1η

The electrophilicity index (ω) and nucleophilicity index (ε) are crucial parameters for assessing a molecule’s electron-accepting and electron-donating tendencies, and they are calculated as follows^71^:

(6) ω=μ22η

(7) ε=1ω

Equations (1)–(7) were used to calculate the quantum chemical parameters for chrysin and CFSB (Table 7). Results showed that chrysin possessed higher chemical hardness (*η*) than CFSB, on the other hand, CFSB possessed higher softness (*S*) which suggests higher chemical reactivity for CFSB. Moreover, CFSB demonstrated a lower electrophilicity index (ω), but a higher nucleophilicity index (ε) than chrysin, which indicates that CFSB possesses a higher tendency to donate electrons, thus it is a better nucleophile.

**Table 7.** Calculated quantum chemical parameters for chrysin and CFSB.

Parameter	Chrysin	CFSB
EA (eV)	1.9573	1.8808
IP (eV)	6.3606	5.2610
χ (eV)	4.1590	3.5709
*η* (eV)	2.2017	1.6901
S (eV)	0.4542	0.5917
ω (eV^-1^)	3.9282	3.7724
ε (eV^-1^)	0.2546	0.2651

Soft molecules, such as CFSB, often demonstrate lower HOMO-LUMO energy gaps. The HOMO-LUMO gap (ΔE_gap_) is an essential parameter that aids in assessing a compound’s stability and reactivity, whereby a higher ΔE_gap_ indicates a molecule of higher stability and lower reactivity^77^. DFT calculations revealed that chrysin had higher stability and lower reactivity due to its higher ΔE_gap_ of 4.4033 eV, on the other hand, CFSB possessed a lower ΔE_gap_ of 3.3802 eV indicating lower stability and higher reactivity.

Therefore, DFT calculations indicated that CFSB seems to have a more nucleophilic character which means that it is expected to interact with its target receptor by electron donation. Furthermore, CFSB’s higher chemical softness and lower ΔE_gap_ relative to chrysin, indicate its higher reactivity and more efficient binding with the target receptor, which in turn would lead to higher bioactivity.

#### Molecular docking studies

Molecular docking of CFSB with CDK2, EGFR, TrkA, VEGFR, topoisomerase II and tubulin was conducted in order to provide a rationale for the inhibitory activity of the compound that was observed against these receptors in the previously discussed *in vitro* assays. These i*n silico* results are expected to provide a plausible explanation behind CFSB’s activity by linking it to its binding affinity.

**Figure 11.**
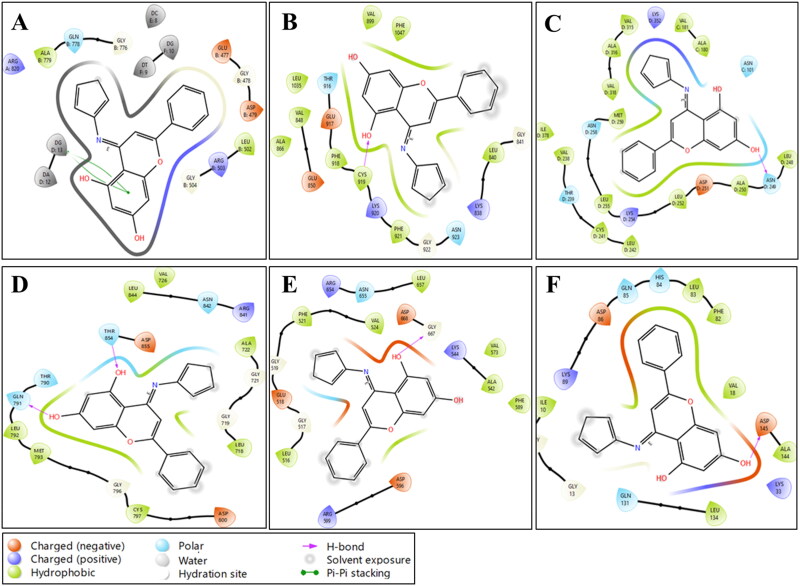
2D images for docking interactions of CFSB with (a) Topoisomerase II; (b) VEGFR; (c) Tubulin; (d) EGFR; (e) TrkA; (f) CDK2.

The previously optimised structure of CFSB was used for docking and the results were summarised in Table 8, while receptor-ligand interactions were visualised in Figure 11. It can be observed that *in silico* studies were generally in agreement with the *in vitro* assay results, which further validates our docking protocol. The binding affinity of CFSB with topoisomerase II was found to be the highest (-10.180 kcal/mol), and it is higher than that of CFSB with the other proteins by a wide margin. This is clearly in line with the *in vitro* assay results, whereby CFSB was found to exert its most potent inhibitory activity against topoisomerase II. This strong binding affinity seems to arise from several interactions such as the π…. π stacking interactions that form between CFSB’s ring C and deoxyguanine (DG 13) of the nucleic acid that is present at the enzyme, in addition to the hydrophobic interactions between ring B and GLY 478. Moreover, docking results revealed that the ferrocenyl moiety of CFSB participates in the binding process by interacting with deoxythymine (DT 9) of the nucleic acid *via* C-H…. π interactions. The ferrocenyl moiety also forms a weak interaction with ARG 820 of topoisomerase II chain B, and this further highlights the crucial role of this moiety in enhancing CFSB’s binding affinity to the protein.

**Table 8.** Selected binding interactions and energies of CFSB and corresponding reference drugs against CDK2, EGFR, TrkA, VEGFR, topoisomerase II and tubulin.

Protein	PDB ID	Ligand	Binding energy (kcal/mol)	Interaction	Interacting residue	Distance (Å)
Topoisomerase II	3QX3	CFSB	−10.180	π…. π	DG 13	4.14, 4.58
				π….alkyl	DT 9	2.49, 2.59
				Hydrophobic	GLY 478	2.33
		Etoposide	−12.239	H-bond	DG 13	2.10
				π…. π	DG 13	3.58, 3.89
				H-bond	GLN 778	2.20
				H-bond	ASP 479	1.72
VEGFR	3B8Q	CFSB	−7.774	H-bond	CYS 919	1.83
				π….alkyl	PHE 1047	2.49, 2.55
		Erlotinib	−9.912	H-bond	CYS 919	1.96
				H-bond	ASN 923	2.18
				Hydrophobic	VAL 914	2.24
				Hydrophobic	LEU 840	2.41
				Hydrophobic	GLU 917	2.35
Tubulin	1SA0	CFSB	−7.128	H-bond	ASN 249	2.04
				Hydrophobic	ALA 250	2.39
				Hydrophobic	CYS 241	2.89
				Polar	MET 259	2.55
		Combretastatin A4	−8.019	Hydrophobic	LYS 352	2.39
				Hydrophobic	VAL 315	2.40
				Hydrophobic	CYS 241	2.19
EGFR	1XKK	CFSB	−6.889	H-bond	GLN 791	2.68
				H-bond	THR 854	2.78
				π….alkyl	ARG 841	2.28
				Hydrophobic	THR 790	2.53
		Erlotinib	−8.774	H-bond	MET 793	2.42
				Hydrophobic	GLN 791	2.37
TrkA	4AOJ	CFSB	−6.315	H-bond	GLY 667	1.99
				π….alkyl	ASN 655	2.33
		Erlotinib	−5.698	H-bond	TYR 591	2.08
				π…. π	PHE 521	5.43
				Hydrophobic	LEU 516	2.34
CDK2	2A4L	CFSB	−5.628	H-bond	ASP 145	1.60
		Erlotinib	−5.266	H-bond	LYS 89	1.87, 2.09
				Hydrophobic	LYS 89	2.04

Docking of CFSB to tubulin revealed a binding affinity of −7.280 kcal/mol which can be considered to be relatively weak, and this might explain its weaker inhibitory activity against tubulin *in vitro*. This binding affinity is partially due to the hydrogen bond interactions between CFSB’s ring C and ASN 249, in addition to the hydrophobic interactions between ring C and ALA 250, as well as ring B and CYS 241. There were also polar interactions between MET 259 and the ferrocene moiety, which again shows the importance of both the chrysin and ferrocenyl moieties in CFSB’s binding process.

CFSB showed lower binding affinities to EGFR, TrkA and CDK2 receptors, and this is in agreement with the *in vitro* enzymatic assays that were conducted, which showed no significant inhibition against these enzymes by CFSB. CFSB showed the least binding affinity to CDK2 (-5.628 kcal/mol), and this seems to be due to the complete lack of interactions between the enzyme and the ferrocenyl moiety which indicates its significance for binding. CFSB also showed a significant lack of hydrophobic interactions with TrkA, which might have contributed to the compound’s weaker binding to the receptor (-6.315 kcal/mol). On the other hand, EGFR formed hydrophobic interactions and hydrogen bonds, in addition to π….alkyl interactions with the ferrocenyl moiety, which resulted in a relatively higher binding affinity for CFSB (-6.889 kcla/mol), but that was still less than that of tubulin. VEGFR is the only receptor which showed relatively stronger binding with CFSB *in silico* (-7.774 kcal/mol), but at the same time no significant inhibitory activity was observed by CFSB against the protein.

Finally, the reference drugs that were used in the *in vitro* enzymatic assays were also docked against their respective targets and results were summarised in Table 8. In most cases, the reference drugs showed higher binding energies with the target proteins than CFSB, which is in line with our *in vitro* results that showed higher enzymatic inhibition for the reference drugs relative to CFSB. TrkA and CDK2 were the only exception, as they showed slightly lower binding energies for erlotinib, but in general it is clearly evident that the *in silico* data is in agreement with the *in vitro* results.

Therefore, in general, molecular docking studies corroborated the results of the *in vitro* assays that were performed on the proteins, whereby generally higher binding affinity *in silico* correlated with higher activity *in vitro*. Moreover, docking studies also highlighted the crucial role that is played by CFSB’s ferrocenyl moiety in enhancing binding to the receptor which in turn would increase the compound’s inhibitory activity.

#### Molecular dynamics (MD) simulations

Molecular dynamics (MD) simulations were performed in order to further analyse the stability and dynamics of the CFSB-topoisomerase II complex and compare it with that of the co-crystallised etoposide-topoisomerase II complex. MD simulations were performed on topoisomerase II as it was the enzyme that got inhibited most potently by CFSB.

**Figure 12.**
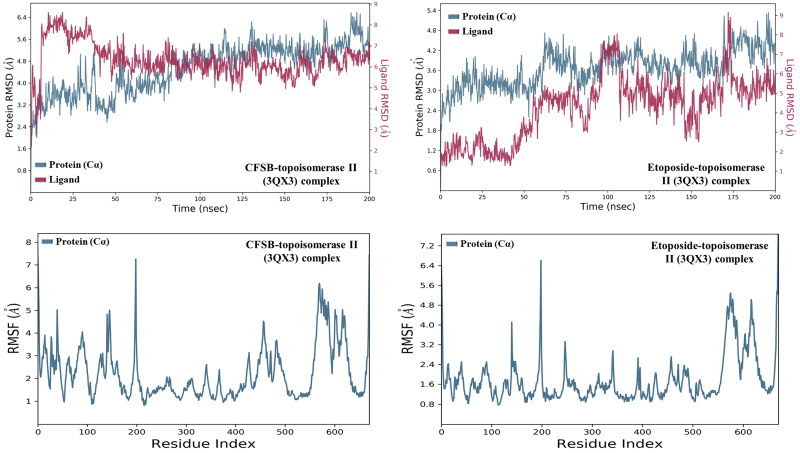
Root mean square deviation (RMSD) and Root mean square fluctuation (RMSF) of CFSB-topoisomerase II and etoposide-topoisomerase II complexes. Protein data were depicted in blue and that of the respective ligands were depicted in maroon.

Figure 12 shows the evolution of root mean square deviation (RMSD) and root mean square fluctuation (RMSF) values for the C-alpha atoms of topoisomerase II complexed with CFSB or etoposide over a duration of 200 ns. The RMSD graph for CFSB-topoisomerase II complex shows the structural stability of the protein over the course of the simulation. In this graph, the protein RMSD starts at a relatively low value (around 1.6 Å**)** and gradually increases over time. After an initial rise, the protein RMSD stabilises around 3.5 to 4.5 Å for 50 ns. This suggests an initial structural adaptation before reaching equilibrium. However, as the simulation progresses beyond 150 ns, the protein RMSD begins to rise slightly, reaching approximately 5.5–6.5 Å by the end. This increase could indicate localised structural adjustments or increased flexibility in specific domains of the protein. Such fluctuations are not uncommon in molecular dynamics simulations and may reflect natural dynamics of the protein, especially in loop regions or flexible domains. Overall, the protein RMSD suggests that the protein remains structurally stable, with some flexibility in specific regions. On the other hand, CFSB’s RMSD initially starts at less than ∼3.0 Å, but peaks to ∼8.0 Å after 20 ns indicating extensive rearrangement. However, the ligand’s RMSD then starts gradually decreasing until it aligns and converges with a stable RMSD of 6 Å until the end of the simulation. This behaviour suggests that while the ligand remains bound, it experiences conformational flexibility, possibly exploring different binding poses within the pocket. As for the etoposide-topoisomerase II complex, the graph shows that the protein’s RMSD starts below 2.0 Å and increases, stabilising around 3.0–4.0 Å after ∼50 ns. There are fluctuations throughout the simulation, which suggest moderate structural flexibility. Notably, towards the end of the trajectory, there are spikes reaching above 4.5 Å, indicating transient conformational shifts. This behaviour may reflect slight domain movements or loop flexibility but remains within an acceptable range for a stable protein-ligand complex. However, etoposide’s RMSD starts below 1.5 Å, indicating a stable binding pose. After ∼50 ns, a gradual increase is observed, reaching ∼3.0 Å, suggesting potential ligand repositioning. Around 80 ns, a further increase is noted, peaking at ∼9.0 Å at about 175 ns, indicating possible partial dissociation or significant rearrangement. Therefore, results of the simulation showed that CFSB and etoposide complexes had comparable stability profiles, however, it seems that etoposide experienced more extensive rearrangement or even possible partial dissociation at the binding site.

RMSF analysis for the CFSB-topoisomerase II complex provides insights into the flexibility of individual residues throughout the simulation. Most residues exhibit low fluctuations (∼1.0 to 2.5 Å), indicating high structural stability, particularly in well-defined secondary structure elements such as alpha-helices and beta-sheets. However, certain residues show higher fluctuations, particularly around residue 200 (∼7.0 Å) and residue 600 (∼6.5 Å). These peaks suggest localised flexibility, which could be due to loop movements, ligand-induced conformational adjustments, or intrinsic disorder in specific regions. The C-terminal residues also display notable fluctuations, which is typical since protein termini are often solvent-exposed and flexible. If these highly flexible regions correspond to binding sites, they may play a crucial role in ligand accommodation or functional conformational shifts. Overall, the RMSF profile indicates a structurally stable protein core with dynamic peripheral regions, which may be essential for the protein’s function and interactions. On the other hand, the etoposide-topoisomerase II RMSF plot reveals overall structural stability, with most residues exhibiting low fluctuations (∼0.8–2.0 Å), suggesting a well-folded core. However, a notable peak around residue ∼200 (∼6.4 Å) and increased flexibility beyond residue ∼500 (4.0–5.0 Å) indicate dynamic regions, likely corresponding to loops or ligand-binding sites. These fluctuations suggest that etoposide may induce local conformational changes or interact with inherently flexible regions of topoisomerase II, potentially influencing its function. The lower RMSF in other regions implies a stable binding environment, while the flexible residues could be crucial for ligand recognition, conformational adaptability, or regulatory mechanisms. Therefore, it seems that for both complexes, topoisomerase II seems to possess good stability, with the exception of some residues where flexibility is more evident and it is suggested that these flexible residues are crucial to accommodate the ligands to provide optimal protein-ligand interactions.

**Figure 13.**
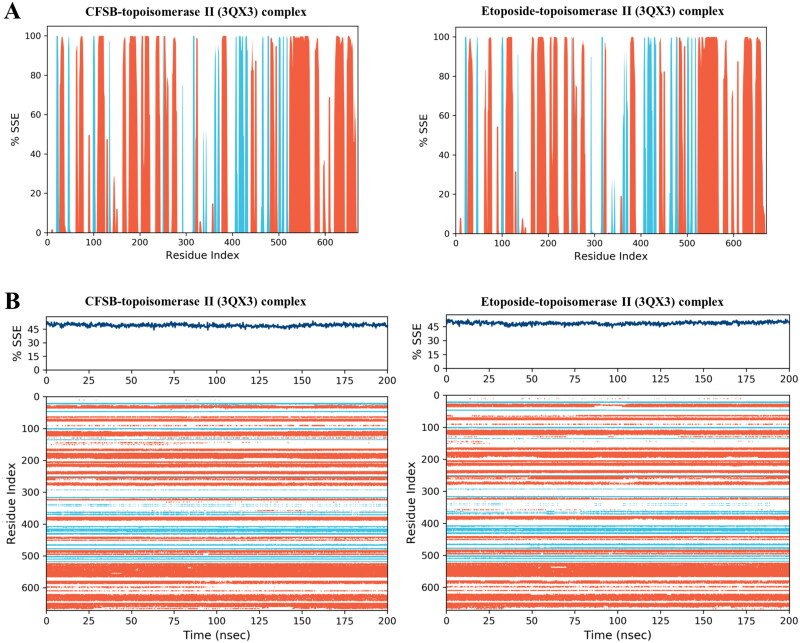
(a) Secondary structure elements (SSE) histogram showing distribution of protein secondary structural elements by residue index; (b) SSE timeline showing evolution of secondary structure over the 200 ns simulation time and participation of different secondary structure residues. Alpha helices were depicted in red and beta-strands in light blue.

Secondary structure elements (SSE) histogram and timeline provide a view of structural retention over the simulation. In the case of CFSB-topoisomerase II complex (Figure 13), the total secondary structure content remains stable (49.18%), with 37.98% helices and 11.19% beta-strands. This suggests that the protein retains its native fold and does not undergo significant unfolding during the simulation. Minor structural transitions in loops may occur but do not compromise the global fold of the protein. Moreover, the SSE timeline for the complex over 200 ns shows a relatively stable secondary structure throughout the simulation, whereby the overall SSE percentage remains around 45%, suggesting consistent structural integrity during the simulation. The colour-coded representation in the SSE timeline shows distinct patterns of alpha-helices (red) and beta-strands (blue) distributed across various residues. The persistence of red regions indicates stable helices and consistent blue bands reflect stable beta-sheet regions while some white gaps suggest transient or unstructured regions. Overall, the structural stability of the complex appears robust, with minimal structural fluctuations observed during the 200 ns timescale. A similar case was also observed with regards to the etoposide-topoisomerase II complex (Figure 13), whereby the consistent presence of helices and strands across the residue index suggests that the protein maintains a well-defined secondary structure. However, some regions exhibit gaps, likely corresponding to flexible loops or disordered segments, which could be functionally significant, especially if they are part of the ligand-binding pocket. The SSE timeline further validates the secondary structure stability for the etoposide complex over the simulation period (200 ns), as the SSE percentage remains steady around 45%, indicating a structurally stable protein. SSE timeline also showed sustained alpha-helices and beta-strands with minimal transitions, reinforcing the stability of structured regions. Minor fluctuations in certain residues, particularly in loop regions, suggest localised flexibility, potentially influenced by ligand binding or intrinsic dynamic behaviour, which may play a role in the functional adaptation of topoisomerase II.

**Figure 14.**
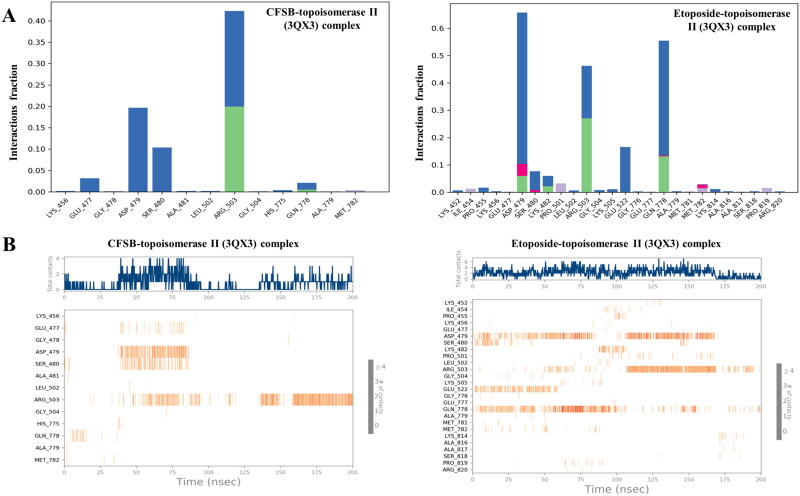
(a) Histogram of protein-ligand interactions during the molecular dynamics simulation. The protein amino acids involved in the interactions were shown on the x-axis. Blue bars refer to water bridges, pink bars to ionic interactions, green bars to hydrogen bonds and light purple bars to hydrophobic interactions; (b) Number of protein-ligand contacts made by the amino acid residues of topoisomerase II binding site. The orange colour refers to the maximum number of contacts and white colour indicates the absence of contacts.

The nature and number of protein-ligand interactions throughout the 200 ns simulation were analysed in Figure 14. In the case of CFSB-topoisomerase II complex, the protein-ligand contacts histogram showed the prevalence of water bridges (blue bars) with dominant interactions in residues like ARG_503, ASP_479, SER_480, and GLU_477, suggesting water molecules play a significant role in stabilising the ligand-protein interface. As for hydrogen bond interactions (green bars), ARG_503 and GLN_778 were found to be the main residues that form such interactions. These bonds enhance ligand specificity and contribute to stability. There are no evident signals for hydrophobic or ionic interactions in the histogram, suggesting ligand binding is primarily mediated through water bridges and a few key hydrogen bonds. Therefore, it can be deduced that ligand binding might depend primarily on solvent interactions, while the few hydrogen bonds provide specificity with ARG_503 being the dominant anchoring residue, while GLN_778 provides secondary support. Moreover, the protein-ligand interaction timeline shows that total number of contacts fluctuates over time, with a noticeable increase after ∼40 ns reaching around 3–4 contacts, followed by another increase after ∼135 ns reaching around 3 contacts. The interaction timeline also confirms that ARG_503 is the primary stabilising residue, forming consistent interactions throughout the simulation. Secondary residues like ASP_479, SER_480 and GLU_477 participate transiently, while HIS_775 and MET_782 contribute minor interactions. GLN_778 provides additional but less stable support. Overall, CFSB forms more consistent and stronger interactions after 40 ns and 135 ns, primarily mediated by ARG_503, ASP_479, SER_480 and GLU_477, which serve as key anchoring points for ligand binding. It is interesting to note that a similar result was also observed in the case of etoposide-topoisomerase II complex, whereby the protein-ligand interactions were dominated by water bridges particularly at ASP_479, GLN_778, ARG_503 and GLU_522, suggesting that water-mediated interactions play a crucial role in stabilising the ligand within the binding pocket. There were also ionic, hydrophobic and hydrogen bond interactions, but their contribution was much less than that of water bridges. ARG_503 was found to form the highest proportion of hydrogen bond interactions followed by GLN_778. Throughout the simulation, ASP_479, ARG_503, and GLN_778 emerge as key residues with persistent interactions. ASP_479 maintains frequent contacts, appearing intermittently but consistently over time, while ARG_503 exhibits strong and continuous interactions, suggesting that it plays a crucial role in ligand stabilisation. GLN_778 shows notable contact persistence, indicating its contribution to ligand retention. This further proves that CFSB and etoposide share a lot of similarities in their binding with topoisomerase II.

**Figure 15.**
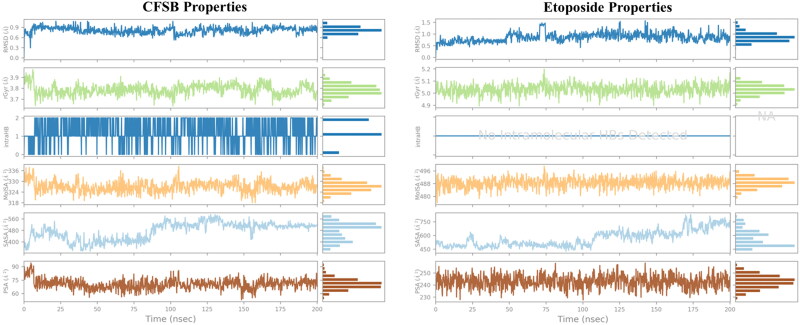
Analysis of different ligand properties during the 200 ns molecular dynamics simulation.

Finally, a further in-depth analysis of the ligands’ properties throughout the 200 ns simulation was conducted and the results have been summarised in Figure 15. CFSB’s RMSD remains below 1.0 Å, indicating that the ligand is relatively stable in the binding pocket. A slight increase towards the end suggests minor conformational adjustments but no significant deviation. Its Radius of Gyration (rGyr) fluctuates around 3.7–3.9 Å, showing that the ligand maintains consistent compactness. No significant unfolding or collapse is observed, suggesting structural stability. The number of intra-ligand hydrogen bonds fluctuates between 1 and 2, suggesting some level of flexibility but without major destabilisation, while Molecular Surface Area (MolSA) values range between 320–340 Å^2^, indicating a stable molecular surface. Solvent Accessible Surface Area (SASA) values range from ∼320 to ∼560 Å^2^, showing gradual exposure to the solvent indicating some level of interaction with the surrounding water molecules, while polar surface area (PSA) remains below 100 Å^2^, meaning limited exposure of polar atoms to the solvent. A relatively lower PSA suggests a good balance between hydrophobic and hydrophilic interactions. Etoposide showed similar ligand properties, except for a lack of intramolecular hydrogen bonds, a higher PSA value (230–260 Å^2^) indicating that the ligand’s polar regions remain solvent-accessible consistently and higher SASA values, especially after 150 ns, reaching to 750 Å^2^ which is indicative of potential ligand repositioning within the binding pocket or transient solvent exposure.

In general, molecular dynamics simulation revealed that the CFSB-topoisomerase II complex is generally stable with the presence of certain flexible regions in the protein in order to accommodate the ligand. CFSB interacted mostly *via* water bridges and hydrogen bonds with the protein primarily *via* the target’s ARG_503 and ASP_479 residues, while the ligand itself was found to be stable with no major conformational changes or dissociation. Finally, it is important to note that the stability profile of CFSB-topoisomerase II is similar to that of etoposide-topoisomerase II, which might partially explain the ligands’ comparable inhibitory activity against topoisomerase II *in vitro*.

#### Molecular mechanics-generalised born surface area (MM-GBSA) analysis

MM-GBSA method was utilised to assess the binding free energy (ΔG_bind_) for every MD simulation. Results of the analysis (Table 9) showed that both CFSB and etoposide complexes had comparable total binding free energies of −17.81 kcal/mol and −27.73 kcal/mol, respectively. Moreover, both complexes were also found to possess comparable or similar binding free energy for most interactions, with etoposide complex being slightly superior. These results corroborate our *in vitro* enzyme inhibition data whereby CFSB and etoposide had comparable inhibitory activity against topoisomerase II.

**Table 9.** Prime MM-GBSA energies for CFSB and etoposide in complex with topoisomerase II.

Complex	ΔG_bind_ Coulomb (kcal/mol)	ΔG_bind_ Covalent (kcal/mol)	ΔG_bind_ Hydrogen bond (kcal/mol)	ΔG_bind_ Lipophilicity (kcal/mol)	ΔG_bind_ Solvation GB (kcal/mol)	ΔG_bind_ vdW (kcal/mol)	Total MM-GBSA ΔG_bind_ (kcal/mol)
CFSB	−5.55	−0.37	−0.14	−12.11	13.51	−13.15	−17.81
Etoposide	−1.83	4.41	−0.37	−24.29	15.63	−21.29	−27.73

#### In silico ADMET and drug-likeness analysis

Drug-likeness and ADMET properties are important parameters in drug discovery. Therefore, we have conducted an *in silico* ADMET study on CFSB to predict its properties. SwissADME was initially used to predict the drug-likeness of CFSB, and it can be observed from Table 10 that the compound seems to have drug-like properties as it fully obeys the Lipinski’s, Veber’s and Egan’s rules with only one violation for Ghose’s rules. This suggests that CFSB would have high bioavailability.

**Table 10.** Physicochemical and drug-likeness properties of CFSB.

Property	Value
Molecular weight	437.27
Log P	3.43
Hydrogen bond acceptors	4
Hydrogen bond donors	2
Total polar surface area (Å^2^)	65.96
Rotatable bonds	4
Lipinski’s rule violations	0
Veber’s rule violations	0
Ghose’s rule violations	1
Egan’s rule violations	0
Muegge’s rule violations	1

**Figure 16.**
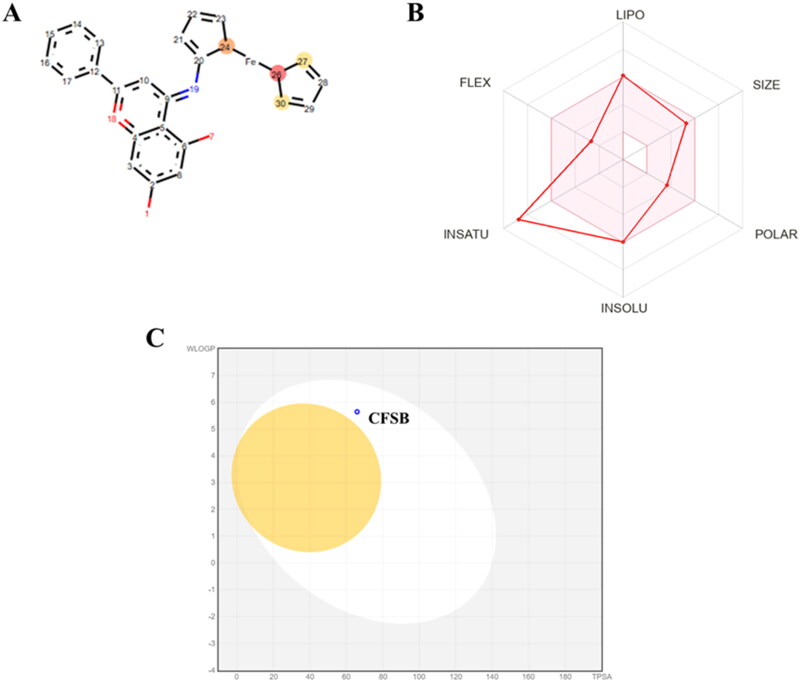
(a) Predicted sites of metabolism in CFSB. Red circle indicates a site with highest probability for metabolism, followed by the orange circle and then the yellow circle. (b) Bioavailability radar chart in which the pink region corresponds to the area that is ideal for oral bioavailability, while the solid red line refers to CFSB properties. (c) BOILED-Egg graph for CFSB. The yellow area corresponds to a compound’s ability to passively permeate the BBB, while the white area corresponds to its ability to be passively absorbed by the GI tract. A blue coloured dot indicates that compound would be easily effluated from the central nervous system by P-glycoprotein.

Further pharmacokinetics analysis using SwissADME and pkCSM (Table 11) revealed that CFSB is expected to be readily absorbed into the GI tract, however, it will not cross the blood-brain barrier (BBB) which suggests high bioavailability and low neurotoxicity, respectively. The compound is predicated to lack mutagenic effects as per the Ames test, and is suggested to also lack any side-effects on the skin, moreover, absence of hERG I inhibition indicates that CFSB will have less cardiotoxic effects. However, some cardiotoxic effects might occur during treatment due to the inhibition of hERG II, in addition, there is a possibility of hepatotoxic side-effects which means that special consideration should be given to these side-effects when determining the appropriate treatment dose and duration. As for CFSB’s metabolism, it is predicted that it would not inhibit the 2 main CYP 450 isoforms; CYP3A4 and CYP2D6, suggesting that the compound would not cause significant drug-drug interactions which could lead to toxic side-effects. PredPS is a software that predicts metabolic stability of a compound in human plasma, whereby a drug is deemed stable if ≥ 85% of it remains at 3 h, and the software outputs the stability result as a probability, whereby a score of < 0.5 is considered stable. Accordingly, CFSB was found to have a 0.09 probability which suggests that it will be stable in human plasma. Finally, SMARTCyp was used to predict the most likely sites on CFSB that will undergo metabolism (Figure 16(a)), and it was found that metabolism is expected to occur mostly on carbons of the cyclopentadiene rings.

**Table 11.** *In silico* pharmacokinetics analysis of CFSB using SwissADME, pkCSM and PredPS.

Property	Value
GI absorption	High
BBB permeation	No
P-gp substrate	Yes
CYP1A2 inhibitor	Yes
CYP2C19 inhibitor	No
CYP2C9 inhibitor	Yes
CYP2D6 inhibitor	No
CYP3A4 inhibitor	No
Metabolic stability in human plasma	0.09 (Stable)
AMES toxicity	No
hERG I inhibitor	No
hERG II inhibitor	Yes
Hepatotoxicity	Yes
Skin sensitisation	No

CFSB’s oral bioavailability was further analysed *in silico*, and the results were displayed on a bioavailability radar chart (Figure 16(b)). The radar chart consisted of six physicochemical properties, namely; Lipophilicity (LIPO), Size, Polarity (POLAR), Insolubility (INSOLU), Insaturation (INSATU) and Flexibility (FLEX). CFSB’s radar chart indicated that it would most probably possess high oral bioavailability as it only violated one related parameter which is insaturation. The BOILED-Egg model (Figure 16(c)) is mainly used to assess a compound’s ability to permeate the blood brain barrier (BBB) and its ability to be adequately absorbed into the GI tract^35^. It can be seen that CFSB (blue dot) lies within the white region of the graph suggesting that it will efficiently be absorbed into the GI tract, and would most probably not lead to any neurotoxicity as it would not be able to cross the BBB. Moreover, CFSB’s blue coloured dot on the graph indicates that it would efficiently be removed from the CNS by P-glycoprotein, which is desirable in this case to avoid any potential side-effects.

Therefore, it is predicted that CFSB would possess desirable ADMET and drug-likeness properties with high bioavailability and metabolic stability, however, treatment doses would have to be adjusted to account for its potential few side-effects.

## Conclusions

In summary, this study utilised the biological activities of chrysin as well as ferrocene, and combined them using an imine group linker which is also bioactive, to result in a highly active molecular hybrid; CFSB. We aimed to highlight the importance of rational-based drug design, specifically molecular hybridisation, in producing effective bioactive agents that are also economic. CFSB’s synthesis was simple and economic, and at the same time, it exerted high bioactivity especially against HepG2. Moreover, the high selectivity index of the compound against cancer cells suggests the presence of less side effects. A key feature of this study is the detailed investigation that was conducted to elucidate CFSB’s mode of action against HepG2. The compound was found to induce G1-phase cell-cycle arrest and apoptosis, in addition to MMP9 expression reduction. Moreover, CFSB was screened against eight different protein targets, out of which only topoisomerase II and tubulin were found to be inhibited by the compound, with topoisomerase II being inhibited at a potency comparable to that of etoposide highlighting its high bioactivity. To further understand the high anti-topoisomerase II activity of CFSB, *in silico* studies, such as DFT calculations, docking, in-depth molecular dynamics simulation, MM-GBSA and ADMET were conducted. This comprehensive study utilised different aspects of drug discovery research, starting from synthesis to detailed cellular as well as enzymatic assays, and finally *in silico* studies. Furthermore, it is expected that this work will shed light on the promising effects of chrysin analogues, and molecular hybridisation as an effective technique for producing highly active molecules in a rational, simple and economic way.

## Supplementary Material

Supplementary_material_Revised2_ Clean.docx

## Data Availability

The datasets presented in the current study are available from the corresponding author upon reasonable request.
