# Supplementary material for: Synthesis, in vitro and in silico studies of a novel chrysin-ferrocene Schiff base with potent anticancer activity via G1 arrest, caspase-dependent apoptosis and inhibition of topoisomerase II
Source: J Enzyme Inhib Med Chem. 2025 May 21;40(1):2501377. doi: 10.1080/14756366.2025.2501377 (PMC12096666; doi:10.1080/14756366.2025.2501377)
Supplement: Supplementary_material_Revised2_ Clean.docx [file IENZ_A_2501377_SM1013.docx]

SUPPLEMENTARY MATERIAL

Synthesis, *in vitro* and *in silico* studies of a novel chrysin-ferrocene Schiff base with potent anticancer activity *via* G1 arrest, caspase-dependent apoptosis and inhibition of topoisomerase II


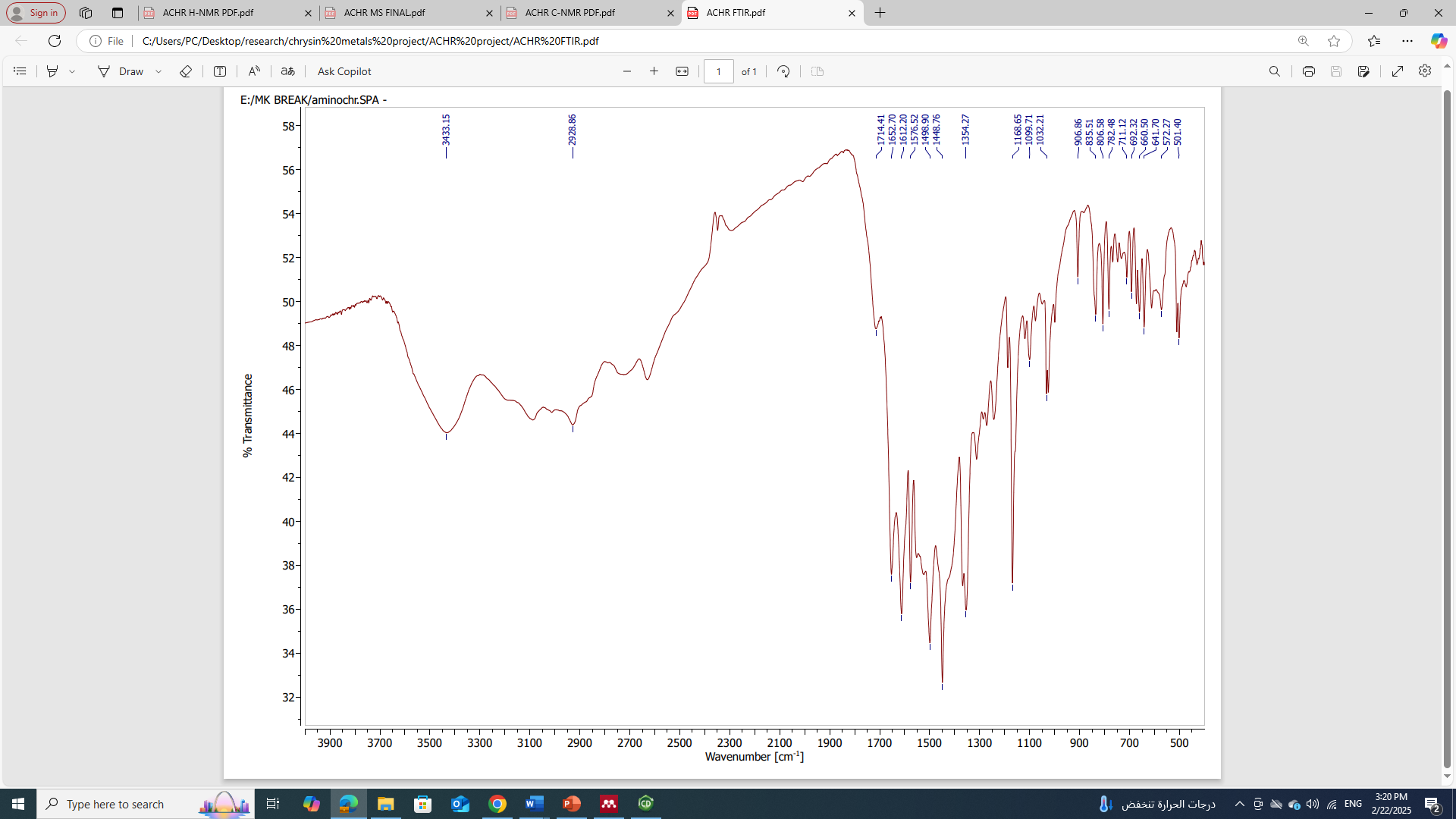


**Figure S1.** FTIR spectrum of CFSB


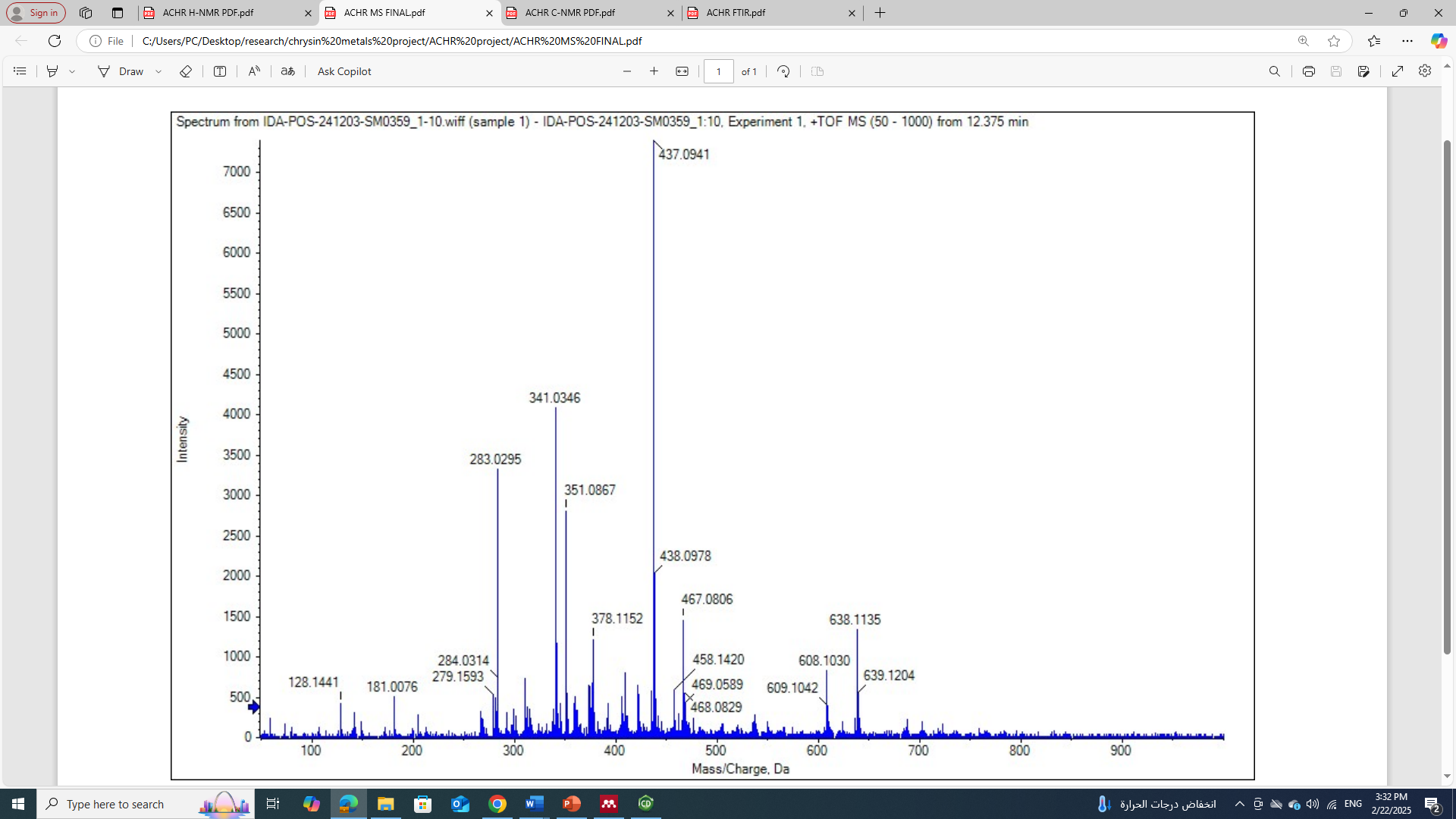


**Figure S2.** HRMS spectrum of CFSB


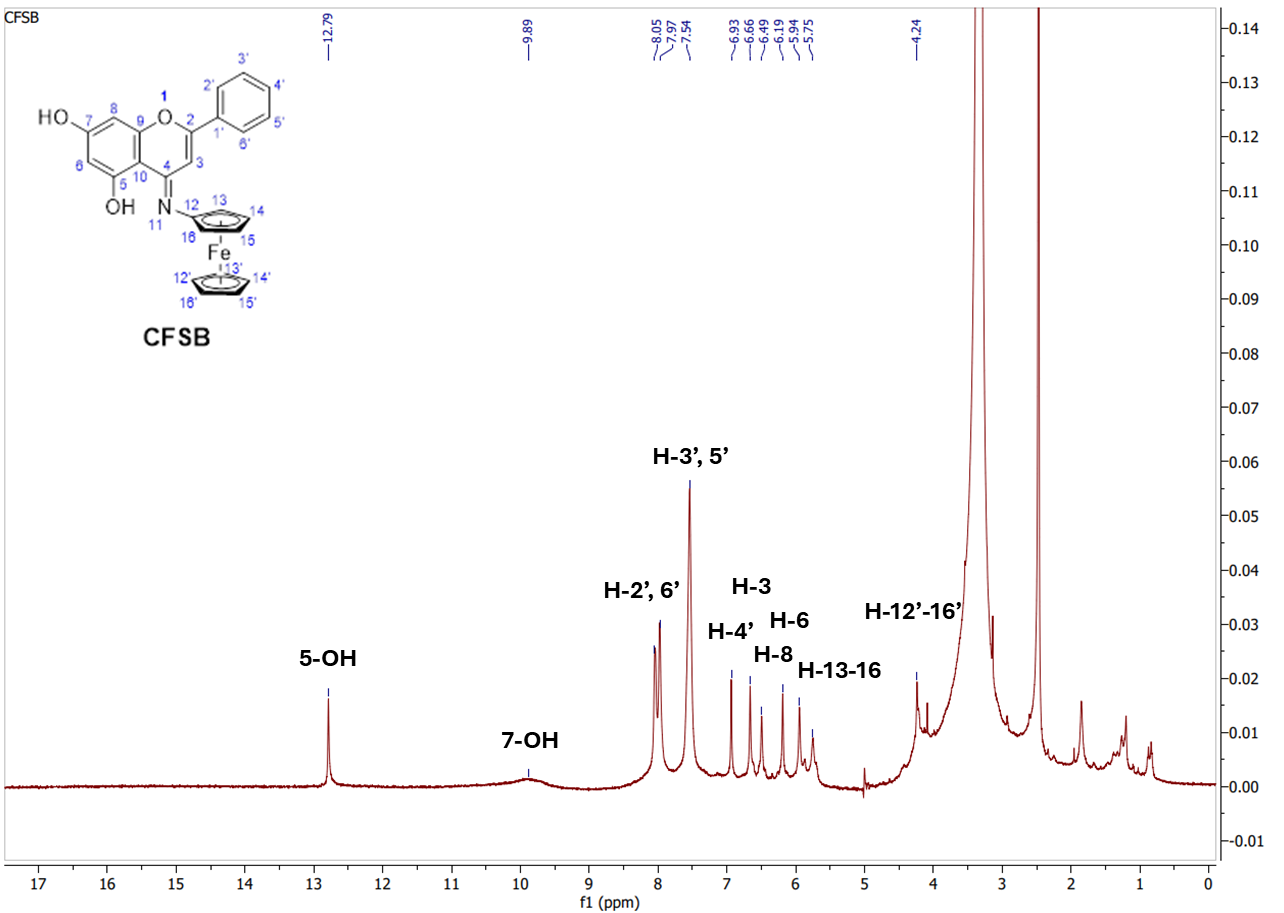


**Figure S3. ^1^**H NMR spectrum of CFSB. Fe (II) paramagnetic effects resulted in peaks that are broad, without clear splitting patterns and of low intensity, especially for nuclei that are nearest to the metal ion.


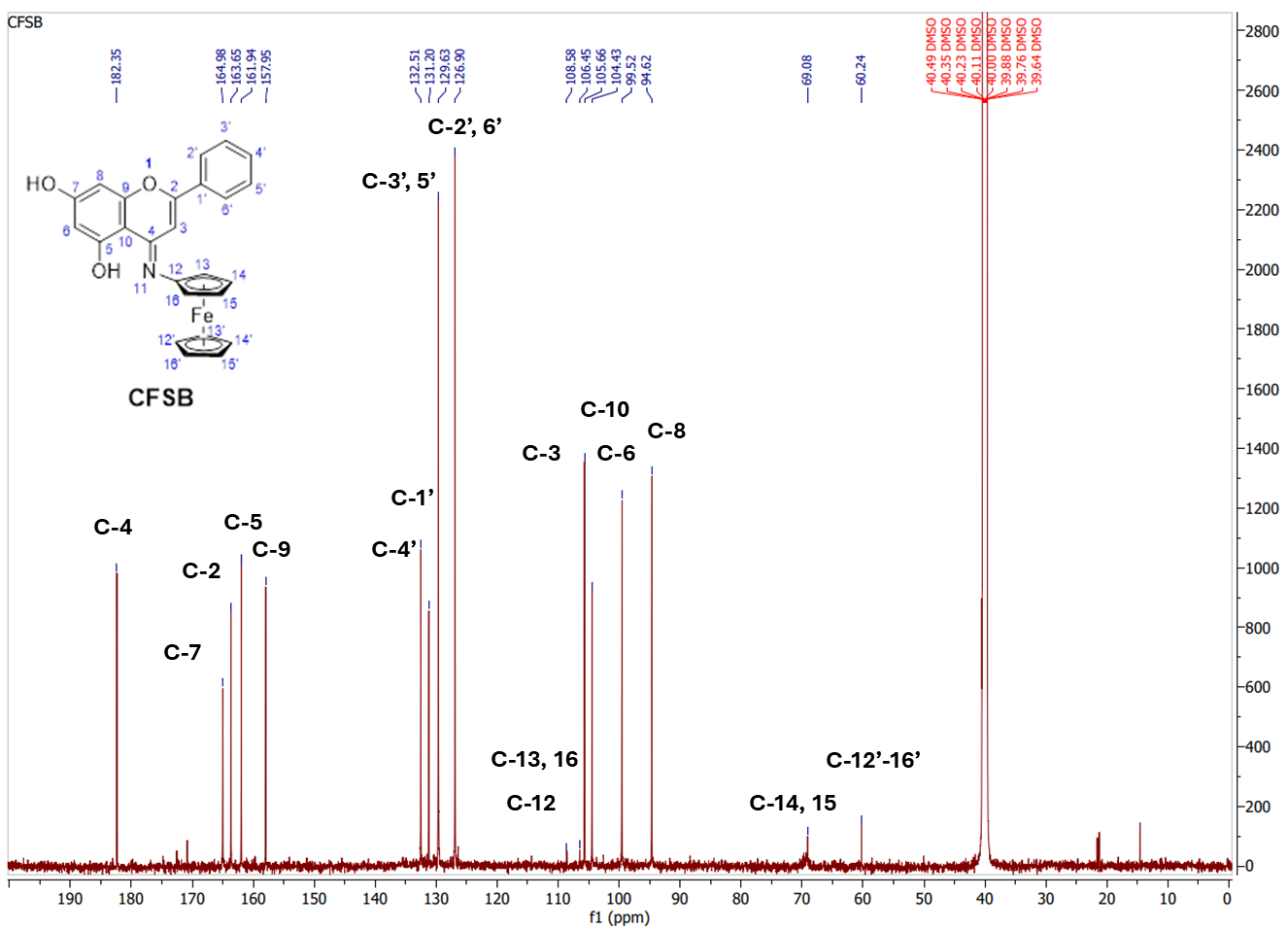


**Figure S4. ^13^**C NMR spectrum of CFSB. Fe (II) paramagnetic effects resulted in peaks of low intensity especially for nuclei that are nearest to the metal ion.


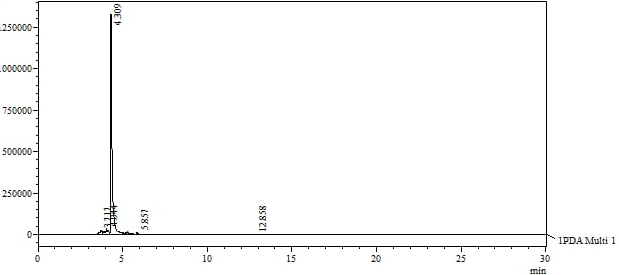


**Figure S5.** HPLC analysis of CFSB


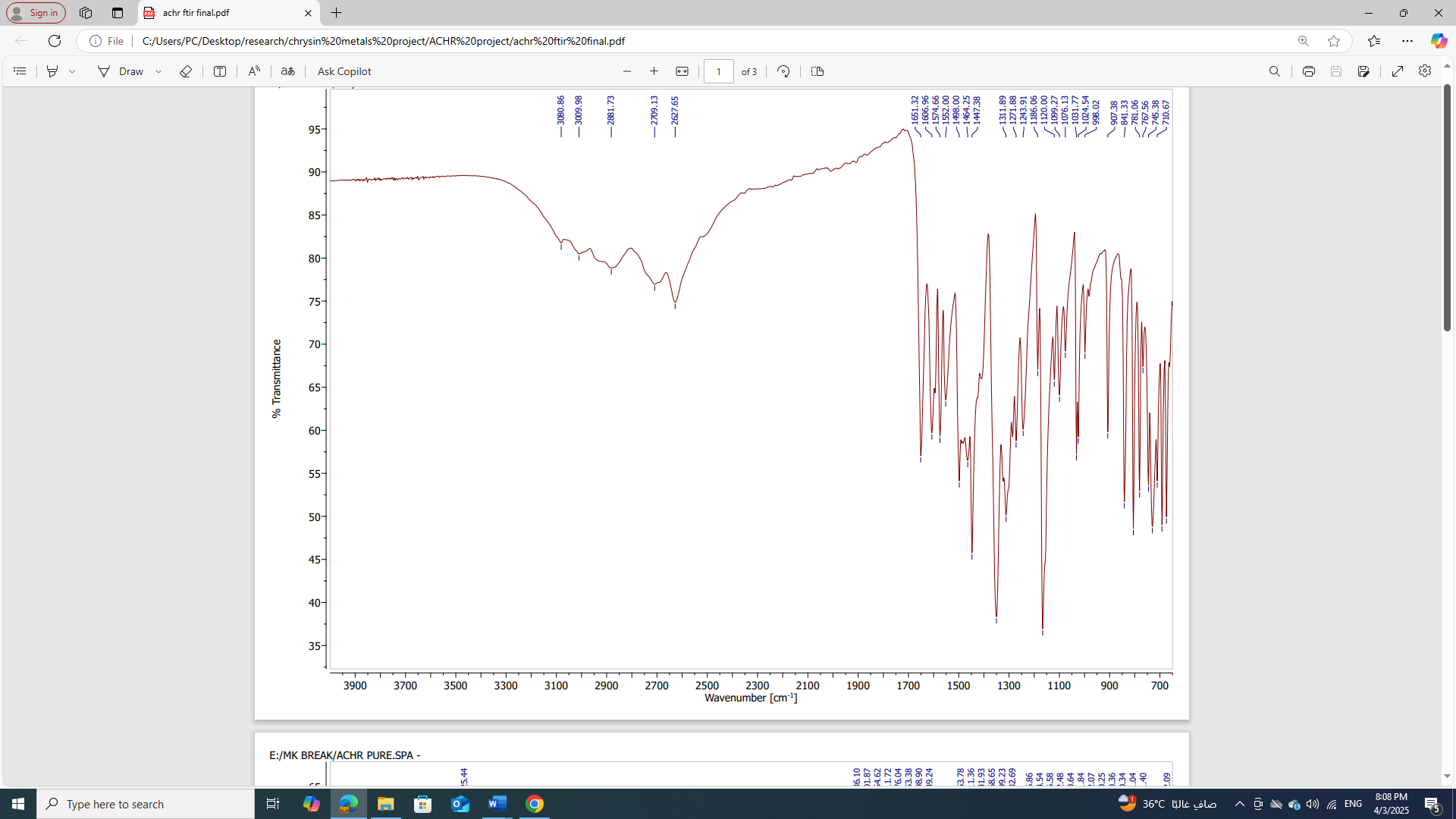


**Figure S6.** FTIR spectrum of chrysin


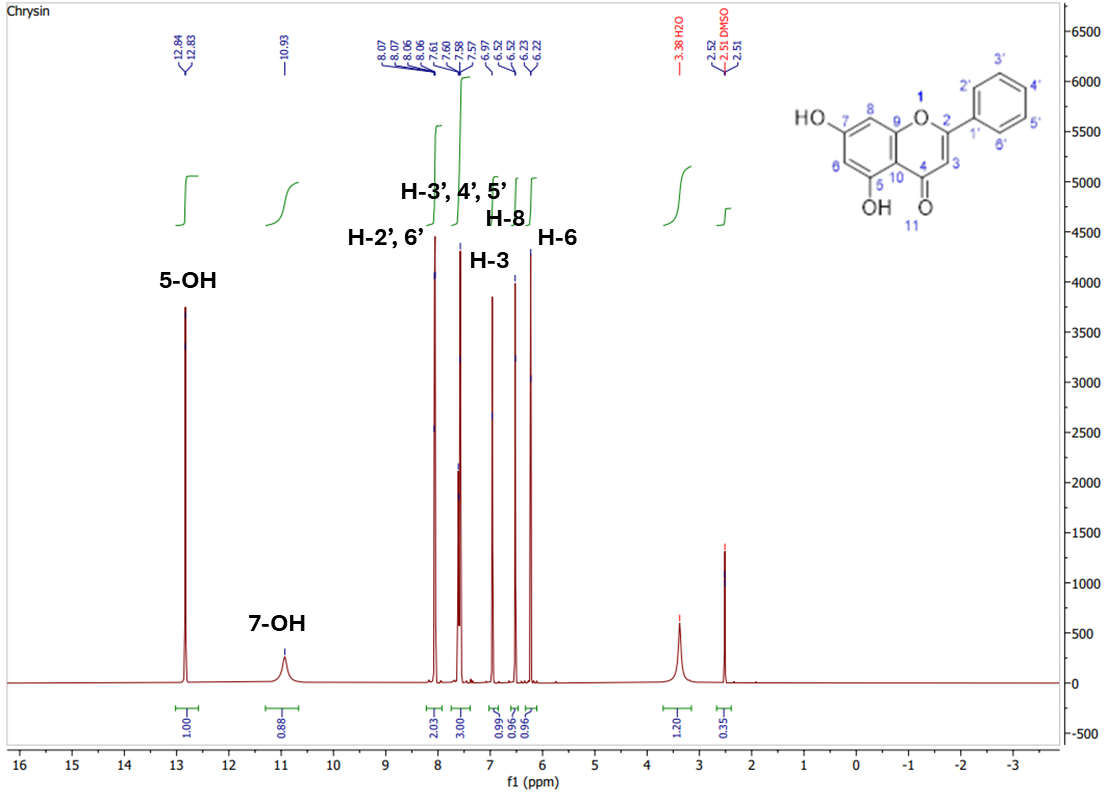


**Figure S7.** H-NMR spectrum of chrysin


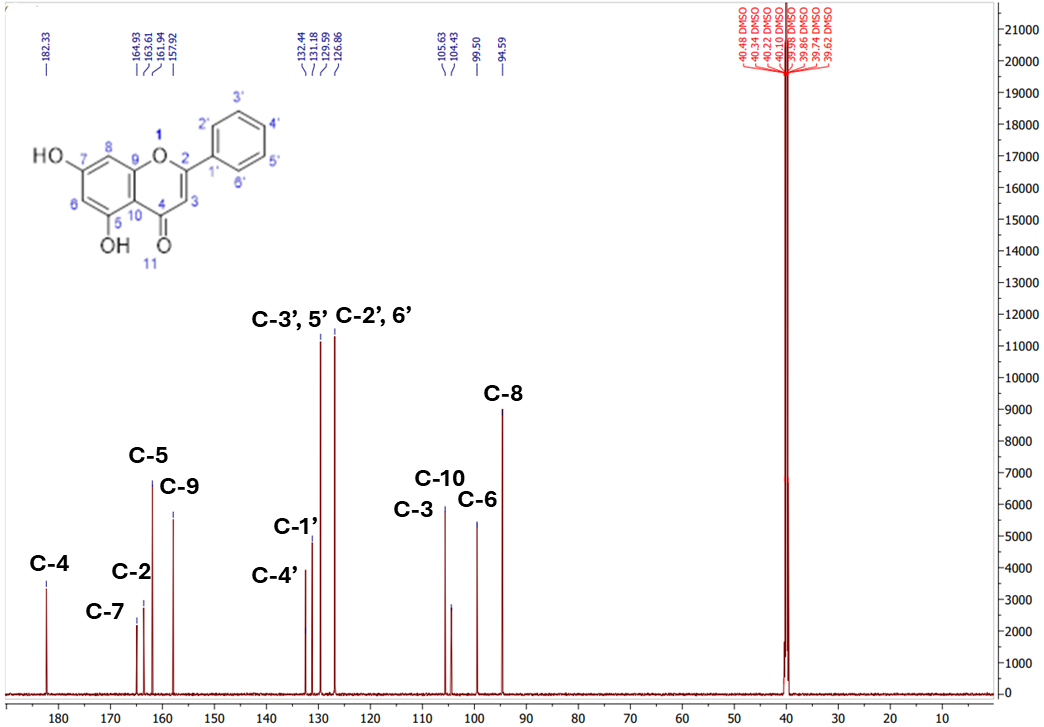


**Figure S8. ^13^**C NMR spectrum of chrysin
